# In *Arabidopsis thaliana* Cd differentially impacts on hormone genetic pathways in the methylation defective *ddc* mutant compared to wild type

**DOI:** 10.1038/s41598-021-90528-5

**Published:** 2021-05-26

**Authors:** Marianna Pacenza, Antonella Muto, Adriana Chiappetta, Lorenzo Mariotti, Emanuela Talarico, Piero Picciarelli, Ernesto Picardi, Leonardo Bruno, Maria Beatrice Bitonti

**Affiliations:** 1 Department of Biology, Ecology and Earth Science, University of Calabria, Arcavacata di Rende, CS Italy; 2 Department of Agriculture, Food and Environment, University of Pisa, Pisa, PI Italy; 3 Department of Biosciences, Biotechnology and Biopharmaceutics, University of Bari, Bari, BA Italy

**Keywords:** Ecology, Plant sciences

## Abstract

DNA methylation plays an important role in modulating plant growth plasticity in response to stress, but mechanisms involved in such control need further investigation. We used *drm1 drm2 cmt3* mutant of *Arabidopsis thaliana,* defective in DNA methylation, to explore metabolic pathways downstream epigenetic modulation under cadmium (Cd) stress. To this aim, a transcriptomic analysis was performed on *ddc* and WT plants exposed to a long-lasting (21 d) Cd treatment (25/50 µM), focusing on hormone genetic pathways. Growth parameters and hormones amount were also estimated. Transcriptomic data and hormone quantification showed that, under prolonged Cd treatment, level and signalling of growth-sustaining hormones (auxins, CKs, GAs) were enhanced and/or maintained, while a decrease was detected for stress-related hormones (JA, ABA, SA), likely as a strategy to avoid the side effects of their long-lasting activation. Such picture was more effective in *ddc* than WT, already at 25 µM Cd, in line with its better growth performance. A tight relationship between methylation status and the modulation of hormone genetic pathways under Cd stress was assessed. We propose that the higher genome plasticity conferred to *ddc* by DNA hypomethylated status underlies its prompt response to modulate hormones genetic pathways and activity and assure a flexible growth.

## Introduction

Plants, as sessile organisms, are under constant influence of environment, which modulates their growth and development through a multiplicity of signals. Therefore, plants evolved molecular mechanisms to sense and rapidly adapt to the wide range of environmental changes occurring at diurnal, seasonal and stochastic level, thus exhibiting high growth plasticity^1^.

A role in such plasticity is paid by epigenetic mechanisms, including DNA methylation, which act on the chromatin status allowing a simultaneous and wide regulation of gene expression. Indeed, studies on both the model *Arabidopsis thaliana* and fruit crops demonstrated that methylome dynamic, beside playing a role at evolution level, is involved in the control of plant ontogenesis and modulate plant response to external cues, including multiple stresses^2^. In particular, either hypermethylation or hypomethylation were detected in plants under different stressors^3^. However, despite all this information, many aspects of the mechanisms that translate the information superimposed by DNA methylation into downstream regulation of gene expression remain still unclear.

A suitable tool to investigate these aspects is provided by *Arabidopsis thaliana* methylation-defective mutants, many of which exhibit phenotype pleiotropic alterations^4,5^. In plants, DNA cytosine methylation occurs in all sequence context and is driven by three enzyme families: the METHYLTRANSFERASES (MET), acting in maintaining methylation in the symmetric CG context, the DOMAINS REARRANGED METHYLTRANSFERASES (DRMs), acting as de novo methyltransferases in the asymmetric CHH context (H = A, C or T) and the plant specific CHROMOMETHYLASES (CMTs), acting primarily in the maintenance of CHG symmetric and CHH asymmetric methylation, but also playing a role in de novo DNA methylation^5,6^. DNA glycosylases contribute to the overall methylome pattern by removing methylated cytosines^5^. In this work, we used the triple *Arabidopsis thaliana drm1 drm2 cmt3* (*ddc*) mutant, combining mutations on *DRM1, DRM2*, and *CMT3* genes*,* defective in both maintenance and de novo DNA methylation^7^. The aim was to investigate molecular and cellular mechanisms that are modulated by DNA methylation in response to stressful factors, such as heavy metal presence.

Heavy metals are naturally present in soils and some of them are required at trace quantities as essential elements. However, at high levels they affect cell homeostasis and are harmful for all organisms^8^. In our study we selected Cd, one of the most harmful and not essential heavy metals, as a stressor due to its ubiquitous presence, long incubation period, ability to migrate and strong ecotoxicity^8^. Moreover, Cd in the soil is easily adsorbed by plants and at high concentration inhibits their growth and development by impacting on several metabolic processes through a wide range of structural and molecular changes, including epigenetic modifications^9^.

On this basis, *Arabidopsis ddc* mutant and wild type (WT) lines were exposed, from germination to 21 d after germination (DAG), to 25 and 50 µM Cd concentrations, whose growth inhibitory effect was previously documented in WT^10^. Plant growth parameters were monitored, and a transcriptome approach was applied to compare how genetic networks and related pathways were affected by Cd in *ddc* mutant compared to WT. The obtained results clearly showed that methylation status is involved in modulating plant response to Cd stress.

## Results

### Plant growth

**Figure 1:**
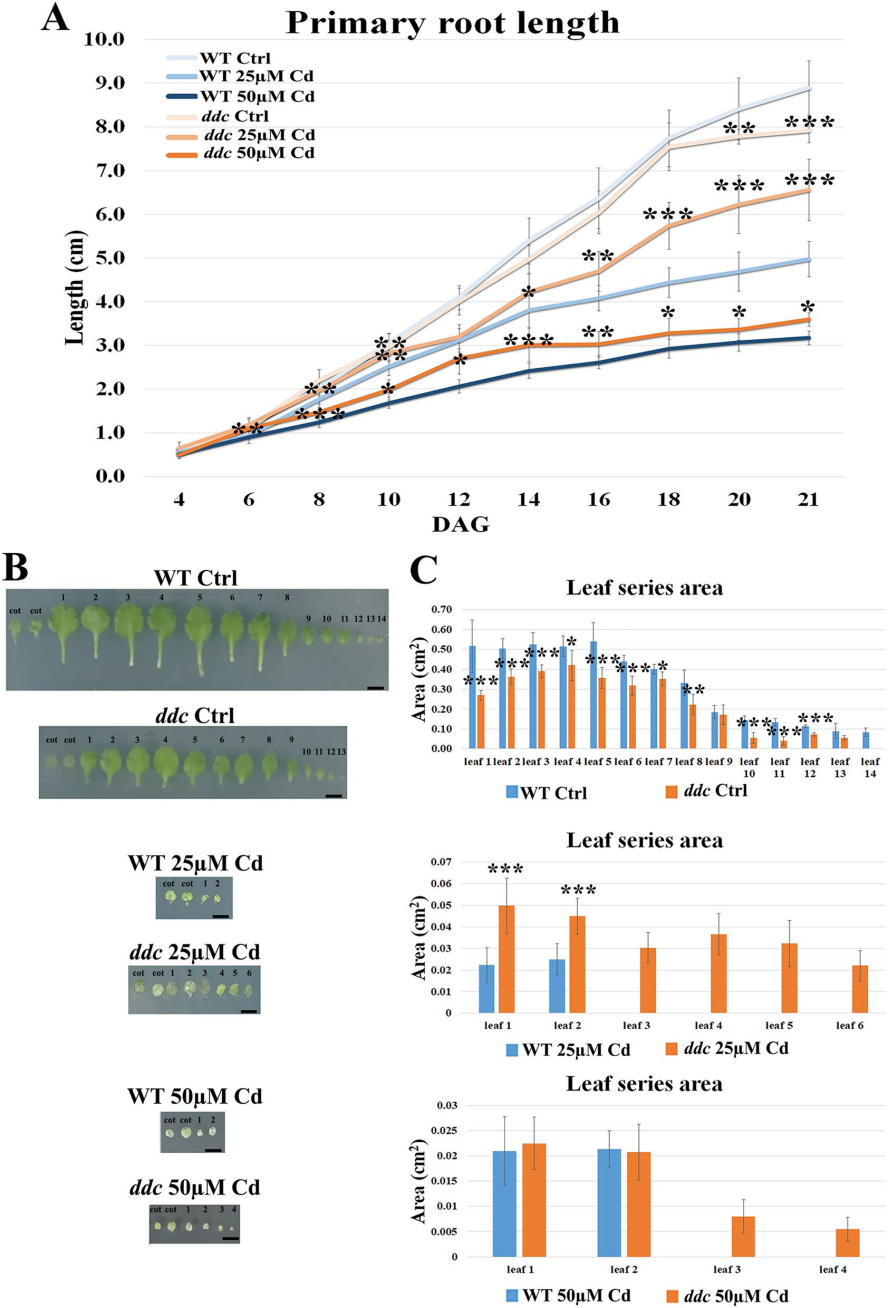
(**A**) Primary root length (**B**) Picture of rosette leaf series and (**C**) rosette leaf area (cm^2^) of WT and *ddc* plants of *A. thaliana*, germinated and grown for 21 DAG in long day condition: (i) on growth medium added with 25 or 50 µM Cd; (ii) on growth medium without Cd as control (Ctrl). Root length was monitored up to 21 days after germination (DAG) every two days from germination. The results represent the mean value (± SD) of three independent biological replicates (n = 45). Asterisks indicate significant pairwise differences using Student’s *t*-test (*P ≤ 0.05; ** P ≤ 0.01; *** P ≤ *0.001)*, performed between *ddc vs* WT subjected to the same treatment. Bars, 0.5 cm.

Primary root length and rosette size were estimated. Control root length, measured until 21 DAG, was lightly minor in *ddc vs* WT (Fig. 1A). Cd differentially inhibited root growth in the two samples: at 21 DAG, 25 and 50 µM Cd-treated roots were 1.2 and 2.2 fold shorter than control roots in *ddc,* while in the WT Cd-treated roots were 1.8 and 2.8 fold shorter than control ones (Fig. 1A). Consequently, at 21 DAG root of Cd-treated samples was longer in *ddc vs* WT, particularly at the lowest Cd concentration.

Rosette size was estimated at 21 DAG, corresponding to the period necessary for its full development^11^, by evaluating leaf number and area. Control plants of both *ddc* and WT exhibited a complete leaf series, although most leaves resulted smaller in *ddc* (Fig. 1B,C). Cd affected rosette development reducing leaf number and area, less in *ddc* than WT, resulting into a higher leaf area and/or number in *ddc* under both Cd concentrations (Fig. 1B,C).

### Gene expression profile

RNA-Seq analysis provided an overview of gene expression profile of Cd-treated and control plants of both *ddc* and WT. The following comparisons were performed**:** *ddc vs* WT under control (Ctrl) conditions (*ddc vs* WT-Ctrl) and 25 and 50 µM Cd treatment (*ddc vs* WT-25 µM Cd; *ddc vs* WT-50 µM Cd); 25/50 µM Cd-treated *vs* Ctrl in *ddc* (25 µM Cd *vs* Ctrl-*ddc*; 50 µM Cd *vs* Ctrl-*ddc*); 25/50 µM Cd-treated *vs* Ctrl in the WT (25 µM Cd *vs* Ctrl-WT; 50 µM Cd *vs* Ctrl-WT).

After DEGs identification (see Supplementary Fig. S1 online) 14 of them were analysed through qRT-PCR to validate transcriptomic analysis (see Supplementary Fig. S2 online). Results were fully consistent with RNA-seq data. Gene Enrichment analysis was also performed, evidencing that Cd strongly impacted on transcriptome in both *ddc* and WT, but in a largely different way (see Supplementary Figs. S3–S9 online). Notwithstanding, a common aspect was that in both *ddc* and WT the genetic pathways (GPs) more impacted by Cd dealt with photosynthesis, stress responses and hormone biosynthesis and signalling.

### Expression pattern of genetic pathways related to hormones

In view of hormones pivotal role in plant development and stress response and considering the assessed epigenetic control on their action and signalling^12^, in this work we analysed in depth how the expression pattern (EP) of hormone-related GPs was modulated in *ddc vs* WT under Cd stress. The most relevant differences are discussed.

### Auxins

**Figure 2:**
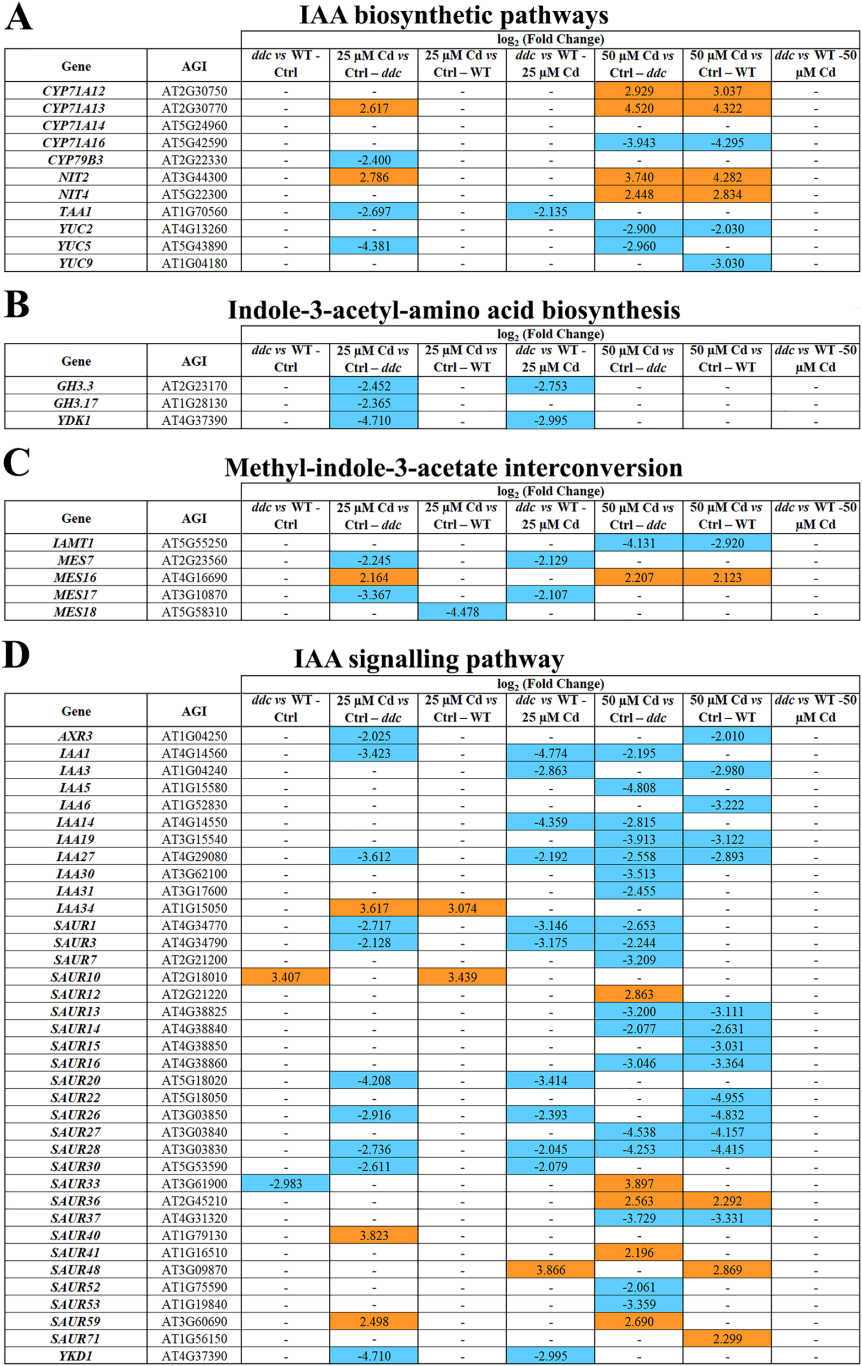
Genes differentially expressed (DEGs) along the pathway of (**A**) auxin biosynthesis, auxin conjugation, (**B**) indole-3-acetyl-amino acid biosynthesis, (**C**) methyl-indole-3-acetate interconversion and (**D**) auxin signalling in *ddc* and WT plants identified through a transcriptomic approach. For each comparison, the log_2_(fold change) of the analysed DEGs was shown in orange and in blue for the upregulated and downregulated genes, respectively. Plants were grown for 21 DAG in long day condition: (i) on growth medium added with 25 or 50 µM Cd; (ii) on growth medium without Cd as control (Ctrl).

Under control conditions, GPs related to auxin biosynthesis showed comparable EP in *ddc* and WT and no DEGs were detected (Fig. 2A). 25 µM Cd induced significant changes only in *ddc* resulting into: i) *TAA1* and *YUC5* downregulation along indole-3-pyruvic acid (IPA) pathway; ii) *CYP71A13* and *NIT2* overexpression along indole-3-acetaldoxime (IAOX) auxiliary pathway, while *CYP79B3* was downexpressed (Fig. 2A). Differently, 50 µM Cd induced similar changes in *ddc* and WT consisting in: (i) a downexpression of *YUC2* along IPA pathway in both samples and *YUC5* and *YUC9* in *ddc* and WT, respectively; (ii) overexpression of *CYP71A12, CYP71A13, NIT2*, *NIT4* and downexpression of *CYP71A16* alon*g* IAOX pathway in *ddc* and WT (Fig. 2A).

Auxin level and homeostasis also depend on its oxidative degradation, conjugation and methylation^13^. Under control conditions, GPs related to auxin conjugation and methylation showed comparable EPs in *ddc* and WT and no DEGs were detected (Fig. 2B,C)**,** but were differentially impacted by Cd, mainly at 25 µM concentration. Namely, at 25 µM Cd several genes related to auxin conjugation (*GH3.3, GH3.17, YDK1*) and methylation (*MES7, MES17*) were downregulated in *ddc*, whereas in WT only *MES18,* involved in methyl-indole-3-acetate production, was downregulated (Fig. 2B,C). At 25 µM Cd most of the above genes were downregulated in *ddc vs* WT (Fig. 2B,C), while at 50 µM Cd only two genes, working in auxin methylation were differentially modulated in *ddc* and WT (Fig. 2B,C).

Under control conditions, GP related to auxin signalling exhibited similar EP in *ddc* and WT (Fig. 2D). Differences were induced by Cd. Namely, at 25 µM Cd, *AUX/IAA* family genes, which acts in signalling repression, were globally downregulated in *ddc,* while in WT the repressor *IAA34* was overexpressed (Fig. 2D). Differently, 50 µM Cd effects on *ddc* and WT were quite similar, dealing with *AUX/IAA* family genes downregulation (Fig. 2D). In *ddc* *vs* WT comparisons, at 25 µM Cd above genes were downregulated, while no differences were found at 50 µM Cd (Fig. 2D). Moreover, and somehow unexpectedly, following 50 µM Cd it was observed in both *ddc* and WT a downregulation of several *SAUR*s members, belonging to a large family of auxin responsive genes, which in turn can also have an impact on auxin pathway^14^ (Fig. 2D). Interestingly, such effect was more pronounced in *ddc* than WT. However, it must be mentioned that, although most of them are induced by auxin, several other hormones and co-factors acts upstream *SAUR* genes, regulating their activity in response to both endogenous stimuli and environmental cues^14^.

In summary, in both *ddc* and WT, Cd induced: i) a downregulation of IPA pathway, which is the main auxin biosynthetic pathway^15^ and a simultaneous upregulation of IAOX auxiliary biosynthetic pathway; ii) an enhancement of hormone signalling. However, in the WT such effects occurred only at 50 μM Cd. Moreover, in *ddc* Cd also induced a downregulation of GPs related to auxin conjugation.

### Cytokinins

**Figure 3:**
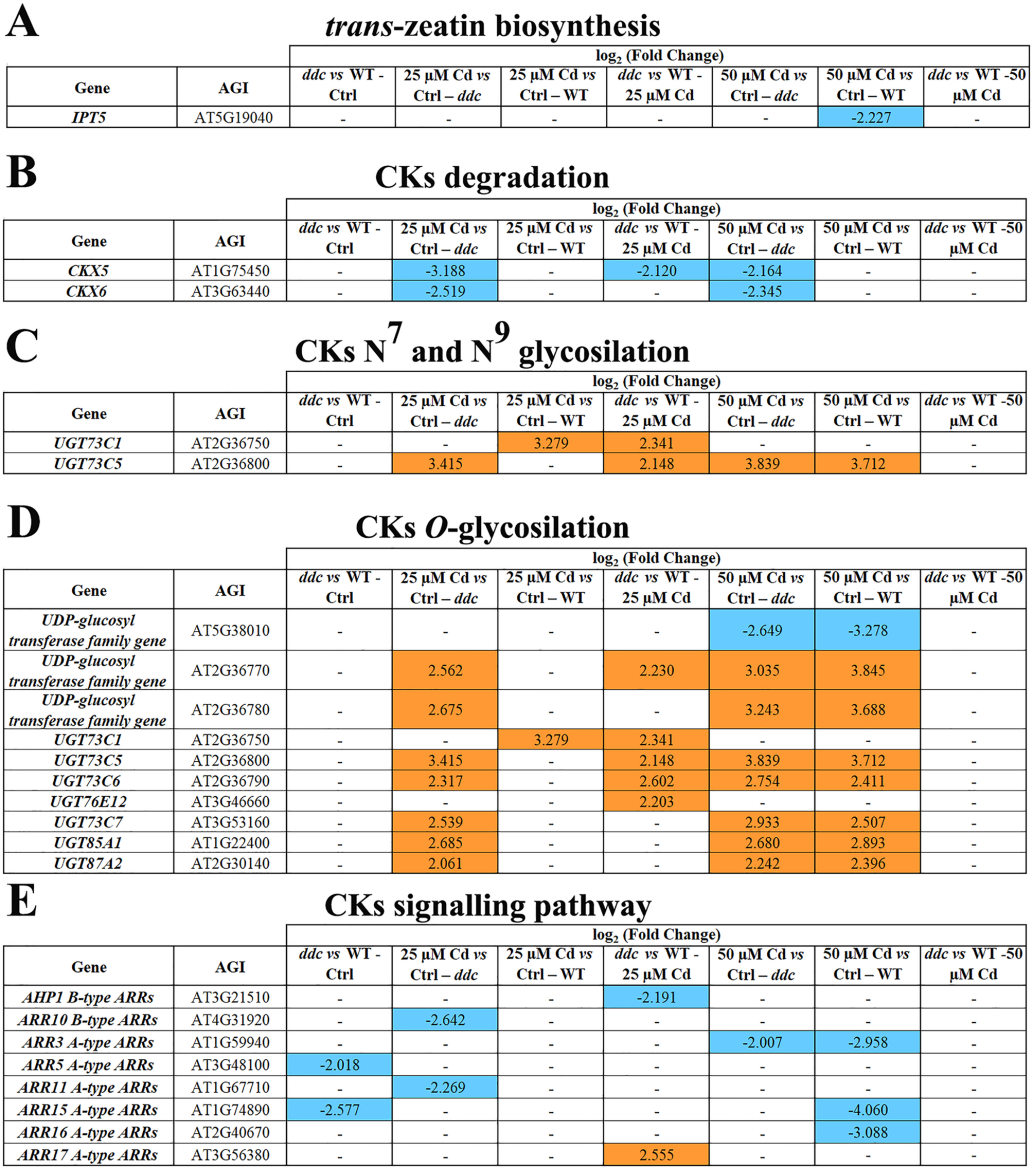
Genes differentially expressed (DEGs) along the pathway of (**A**) *trans-*zeatin biosynthesis, (**B**) CKs degradation, (C) CKs *N*^*7*^*-* and *N*^*9*^*-*glucoside biosynthesis, (**D**) CKs *O-*glycosylation and (**E**) CKs signalling in *ddc* and WT plants identified through a transcriptomic approach. For each comparison, the log_2_(fold change) of the analysed DEGs was shown in orange and in blue for the upregulated and downregulated genes, respectively. Plants were grown for 21 DAG in long day condition: (i) on growth medium added with 25 or 50 µM Cd; (ii) on growth medium without Cd as control (Ctrl).

In all comparisons, GPs related to CKs biosynthesis showed similar EPs, unless for the downregulation in 50 µM Cd-treated WT *vs* Ctrl of *IPT5,* encoding rate-limiting enzyme along the pathway^16^ (Fig. 3A).

Major differences were observed for GPs related to CKs catabolism and conjugation, occurring through cleavage by oxidation and glycosylation, respectively^16^. Under control conditions, these GPs also exhibited similar EPs in *ddc* and WT (Fig. 3B-D). At both 25 and 50 µM Cd a downregulation of *CKX5* and *CKX6,* encoding cytokinin-oxidases, occurred only in *ddc* (Fig. 3B). Differently, Cd impact on GPs related to CKs *N-*glycosylation was almost comparable in *ddc* and WT, resulting into the overexpression of two different genes working in *N*^*7*^*-* and *N*^*9*^*-*glycosylation pathways at 25 µM Cd, and one gene at the higher concentration (Fig. 3C).

Note that at 25 µM Cd the above genes were both overexpressed in *ddc vs* WT, while no differences were found at 50 µM Cd (Fig. 3C). Cd impact on GP related to cytokinin *O-*glycosylation was major, especially in *ddc*, involving at 25 µM Cd the overexpression of seven genes along this pathway compared to Ctrl (Fig. 3D), and one gene in WT 25 µM Cd *vs* Ctrl (Fig. 3D). By contrast, at 50 μM Cd the expression pattern along this pathway was similar in *ddc* and WT, being characterised by the upregulation of the same seven genes above mentioned and the downregulation of *AT5G38010* (Fig. 3D). Finally, at 25 μM Cd, five genes along these pathways resulted upregulated in *ddc vs* WT while a similar EP occurred in *ddc* and WT under 50 μM Cd treatment (Fig. 3D).

Concerning GP involved in CKs signalling, under control conditions *A-ARRs*, encoding negative regulators of CKs signalling^17^, were downregulated and signalling was likely enhanced in *ddc vs* WT (Fig. 3E). 25 µM Cd induced a downregulation of *ARR11 A-type ARRs* and *B-ARR* family *ARR10* transcription factors, which control primary plant response to CKs, only in *ddc* (Fig. 3E). Whereas, at 50 µM Cd both *ddc* and WT showed *A-ARR* downregulation, supposedly leading to pathway upregulation (Fig. 3E). At 25 µM Cd *AHP1,* encoding positive regulators of CKs signalling^18^, was downregulated in *ddc vs* WT, while *ARR17* was overexpressed, suggesting that signalling was downregulated also in *ddc vs* WT (Fig. 3E). No differences occurred between *ddc* and WT at 50 µM Cd (Fig. 3E).

In summary, transcriptomic analysis evidenced that GP related to the biosynthesis of *trans*-zeatin, the most relevant CK, was negatively affected by Cd only in the WT at 50 μM Cd. In response to Cd, GPs related to CKs inactivation were enhanced in both *ddc* and WT, but in *ddc* a downregulation of GP related to CKs cleavage also occurred. Finally, hormone signalling was differentially modulated by Cd in relation to both the sample (*ddc vs* WT) and heavy metal concentration, resulting into a downregulation at 25 µM Cd only in *ddc* and an enhancement in *ddc* and WT at 50 µM Cd.

### Gibberellins

**Figure 4:**
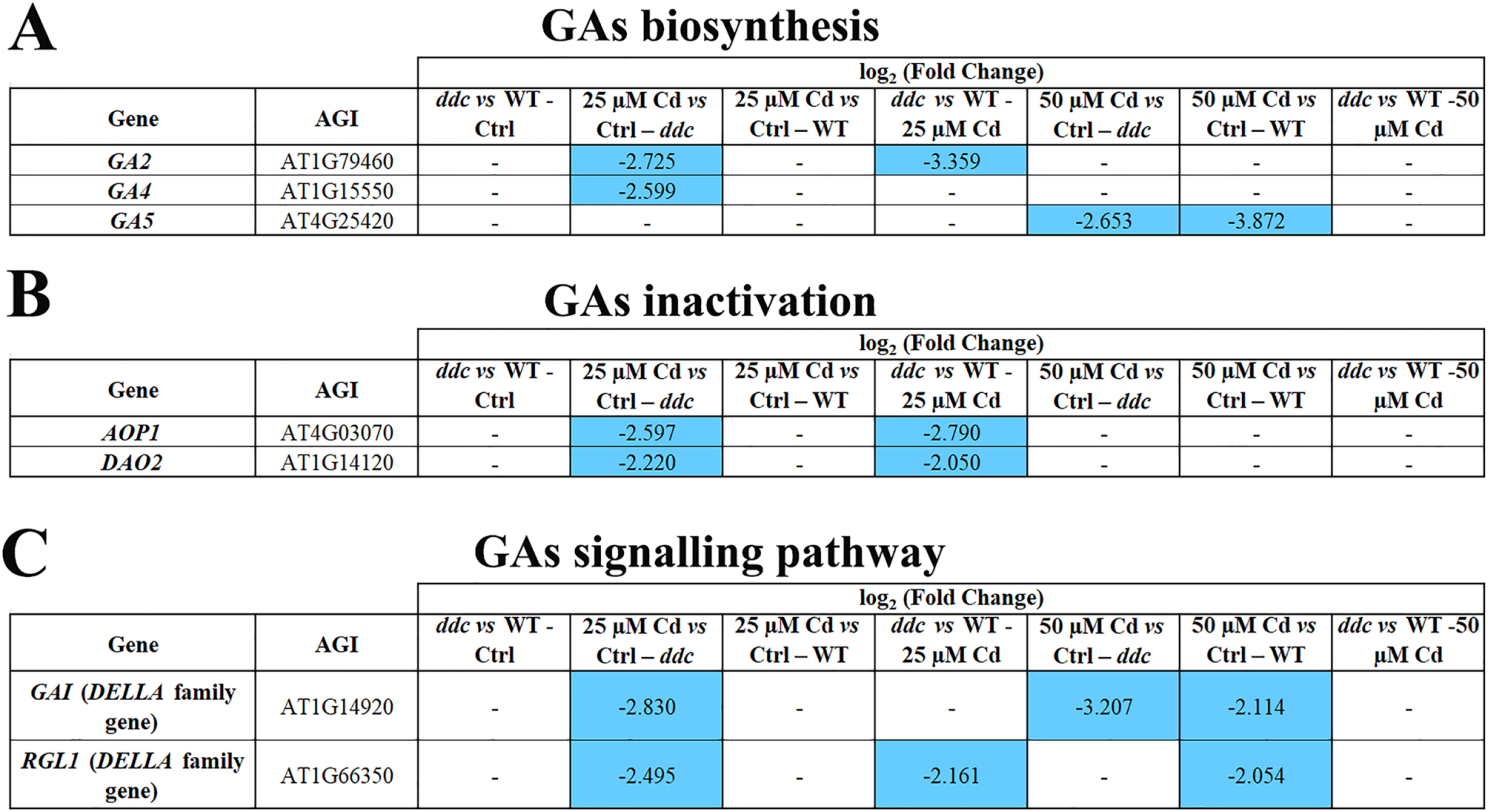
Genes differentially expressed (DEGs) along the pathway of (**A**) GAs biosynthesis, (**B**) GAs inactivation and (**C**) GAs signalling in *ddc* and WT plants identified through a transcriptomic approach. For each comparison, the log_2_(fold change) of the analysed DEGs was shown in orange and in blue for the upregulated and downregulated genes, respectively. Plants were grown for 21 DAG in long day condition: (i) on growth medium added with 25 or 50 µM Cd; (ii) on growth medium without Cd as control (Ctrl).

Under control conditions, GPs related GAs biosynthesis showed similar EPs in *ddc vs* WT (Fig. 4A). 25 μM Cd induced in *ddc*: i) a downregulation of *GA2* encoding the *ent-*kaurene synthase, a pivotal enzyme along the early GAs biosynthetic pathways to synthetize GA_12_; ii) a downregulation of *GA4*, a key gene of GAs biosynthesis, along which bioactive GAs are synthetized^19^ (Fig. 4A). No Cd-induced modulation was observed in the WT (Fig. 4A). On the contrary, at 50 μM Cd both *ddc* and WT showed a downregulation of *GA5* (Fig. 4A). Finally, in *ddc vs* WT the only difference dealt with *GA2* downregulation at 25 µM Cd (Fig. 4A).

GPs controlling GAs inactivation also showed a comparable transcriptional pattern in *ddc* and WT under control conditions, and no DEGs were detected (Fig. 4B). 25 μM Cd induced a downregulation of *DAO2* and *AOP1*, encoding GA2ox enzymes, only in *ddc* (Fig. 4B). Accordingly, in *ddc vs* WT these genes were downregulated only at the lowest Cd concentration (Fig. 4B).

Under control conditions, also GP related to GAs signalling was not differentially modulated in *ddc vs* WT (Fig. 4C). A downregulation of genes encoding DELLA proteins, which act as repressors^20^, was induced only in *ddc* by 25 µM Cd (Fig. 4C) and in both *ddc* and WT at 50 µM Cd (Fig. 4C), suggesting an enhancement of hormone signalling. Consequently, DELLA-codifying genes resulted downregulated also in *ddc vs* WT only at the lowest Cd concentration (Fig. 4C).

In summary, in *ddc* the lowest Cd treatment negatively affected GPs related to GAs biosynthesis but, at the same time, hormone signalling resulted enhanced. In the WT similar effects were observed only at 50 µM Cd.

### Jasmonic acid

**Figure 5:**
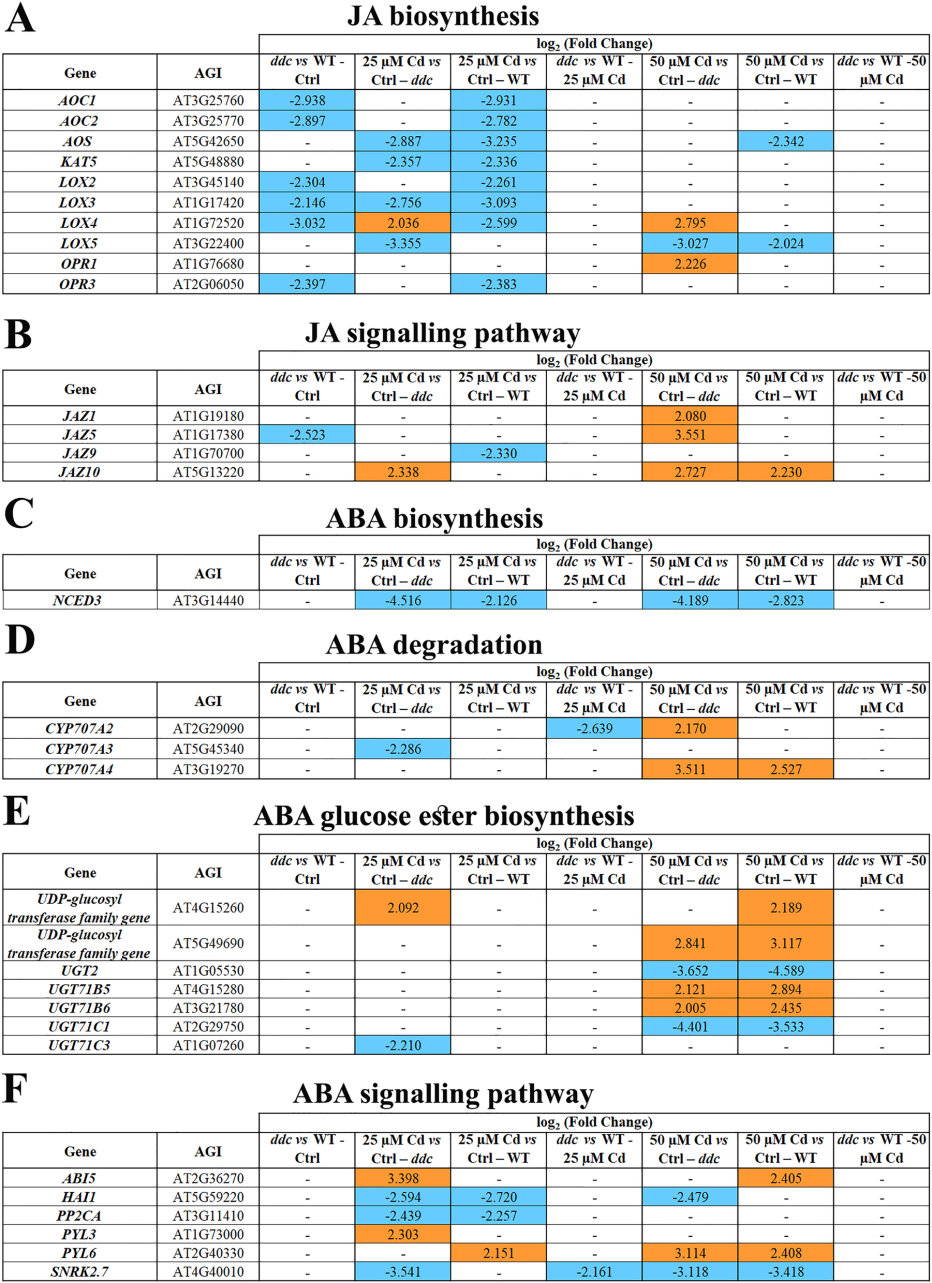
Genes differentially expressed (DEGs) along the pathway of (**A**) JA biosynthesis, (**B**) JA signalling, (**C**) ABA biosynthesis, (**D**) ABA degradation, (**E**) ABA glucose ester biosynthesis and (**F**) ABA signalling in *ddc* and WT plants identified through a transcriptomic approach. For each comparison, the log_2_(fold change) of the analysed DEGs was shown in orange and in blue for the upregulated and downregulated genes, respectively. Plants were grown for 21 DAG in long day condition: (i) on growth medium added with 25 or 50 µM Cd; (ii) on growth medium without Cd as control (Ctrl).

Under control conditions, six genes along the GP related to JA biosynthesis were downregulated in *ddc vs* WT (Fig. 5A). 25 µM Cd induced in *ddc* a downregulation of this GP, except for *LOX4* upregulation (Fig. 5A) and a downregulation involving eight genes in WT (Fig. 5A). At 50 μM, Cd effects were limited to *LOX5* downregulation and *LOX4* and *OPR1* upregulation in *ddc* and *LOX5* and *AOS* downregulation in WT (Fig. 5A). No Cd–induced differences were found in *ddc vs* WT (Fig. 5A).

Concerning the JA signalling-related GP, in control conditions *JAZ5* gene, encoding a protein acting as repressor^21^, was downregulated in *ddc vs* WT, highlighting a signalling enhancement (Fig. 5B). Interestingly, *JAZ10* and *JAZ9* were differentially impacted by 25 µM Cd in *ddc* and WT resulting upregulated and downregulated, respectively. (Fig. 5B). At 50 µM Cd, *JAZ*s were overexpressed in both *ddc* and WT (Fig. 5B). No differences were detected in *ddc vs* WT exposed to Cd (Fig. 5B).

Globally, Cd negatively impacted on the GP related to JA biosynthesis especially in WT. Under Cd treatment hormone signalling was downregulated more in *ddc* than in WT, whatever concentration was applied.

### Abscisic acid

Under control conditions, ABA biosynthesis-related GP showed comparable EP in *ddc* and WT, and no DEGs were detected (Fig. 5C). The only significant Cd effect dealt with *NCED3* downregulation both in *ddc* and WT, regardless of applied concentration (Fig. 5C). Under control conditions, also the GPs related to ABA catabolism showed a comparable EP in *ddc* and WT and no DEGs were detected (Fig. 5D), but Cd differentially impacted on *CYP* genes, involved in phaseic acid degradative production^22^. Namely, at 25 μM Cd, *CYP707A3* was downregulated only in *ddc* (Fig. 5D). Moreover, also *CYP707A2* appeared downregulated in *ddc vs* WT (Fig. 5D). At 50 µM Cd it was observed an upregulation of both *CYP707A2* and *CYP707A4* in *ddc,* and of only *CYP707A4* in WT (Fig. 5D).

Under control conditions, GP related to ABA inactivation through glucose conjugation showed similar EP in *ddc vs* WT (Fig. 5E). 25 μM Cd determined *AT4G15260* upregulation and *UGT71C3* downregulation only in *ddc* (Fig. 5E). At 50 μM, Cd equally impacted on *ddc* and WT, resulting into *UGT71C1* and *UGT2* downregulation, *AT5G49690, UGT71B5, UGT71B6* upregulation and, limited to WT, *AT4G15260* upregulation (Fig. 5E). No differences were highlighted in *ddc vs* WT exposed to Cd (Fig. 5E).

Under control conditions, the GP related to hormone signalling also presented a comparable EP in *ddc* and WT (Fig. 5F). In *ddc,* 25 µM Cd impact on this GP appeared rather complex, resulting in an upregulation of *PYL3*, encoding ABA receptor, and a downregulation of *PP2Cs (PP2CA* and *HAI1)* encoding negative regulators of ABA signalling^23^. Moreover, *ABI5*, codifying a key transcription factor in ABA signalling^24^ belonging to AREBs/ABFs family, was upregulated*.* However, *SnRK2.7* gene, codifying a protein which activate the AREBs/ABFs transcription factors^24^, was downregulated. Based on the prominent role of SnRK2s in plant response to ABA, it is likely that at 25 µM Cd ABA signalling was downregulated in *ddc* (Fig. 5F). Instead, 25 µM Cd determined in WT the upregulation of *PYL6* and the downregulation of *PP2Cs*, suggesting an enhancement of ABA signalling (Fig. 5F). At 50 µM Cd, *PYL6* was upregulated and *SnRK2.7* downregulated in both *ddc* and WT, while *HAI1* was downregulated only in *ddc* (Fig. 5F). When comparing *ddc vs* WT, at 25 µM Cd only *SnRK2.7* was downregulated, while no differences occurred at 50 μM Cd (Fig. 5F).

In summary, Cd determined a slight downregulation of GP related to ABA biosynthesis in both *ddc* and WT regardless of its concentration. ABA catabolic pathway was lightly downregulated in *ddc* at 25 µM Cd but upregulated in both samples at 50 μM Cd. At the transcriptomic level, ABA signalling featured as enhanced in WT and downregulated in *ddc* regardless of Cd concentration.

### Ethylene

**Figure 6:**
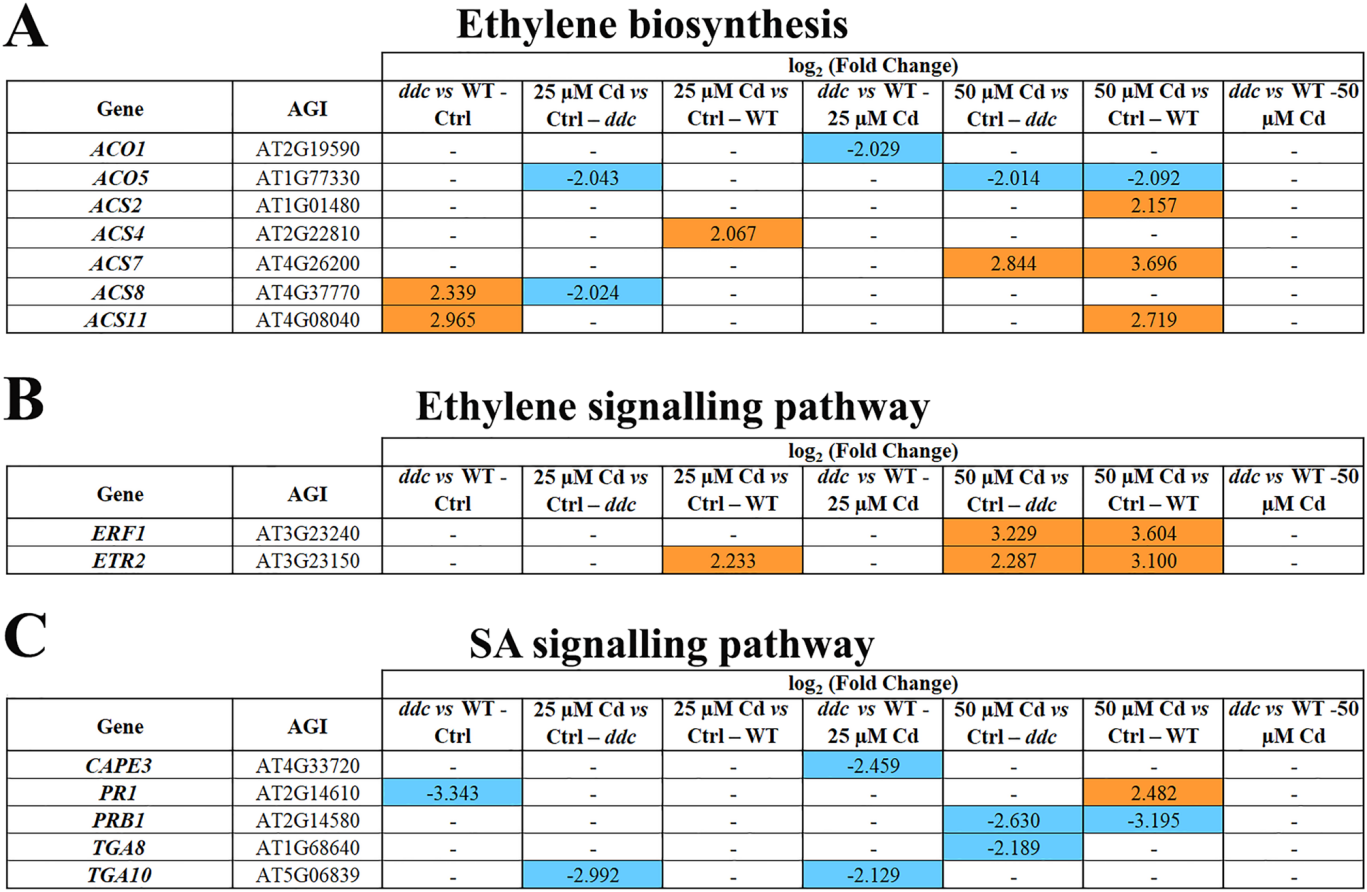
Genes differentially expressed (DEGs) along the pathway of (**A**) ethylene biosynthesis, (**B**) ethylene signalling and (**C**) SA signalling in *ddc* and WT plants identified through a transcriptomic approach. For each comparison, the log_2_(fold change) of the analysed DEGs was shown in orange and in blue for the upregulated and downregulated genes, respectively. Plants were grown for 21 DAG in long day condition: (i) on growth medium added with 25 or 50 µM Cd; (ii) on growth medium without Cd as control (Ctrl).

Along GP related to ethylene biosynthesis, under control conditions *ACS8* and *ACS11* were upregulated in *ddc vs* WT (Fig. 6A). 25 µM Cd determined *ACS8* and *ACO5* downregulation in *ddc* and *ACS4* upregulation in the WT (Fig. 6A). 50 μM Cd induced *ACS7* upregulation and *ACO5* downregulation in both *ddc* and WT and *ACS2* and *ACS11* overexpression only in WT (Fig. 6A). Finally, the only Cd-induced difference in *ddc vs* WT dealt with *ACO1* downregulation at 25 µM Cd (Fig. 6A).

GP related to ethylene signalling showed similar EP in *ddc* and WT both under control conditions (Fig. 6B) and at 25 µM Cd, except for the upregulation of *ETR*2, encoding ethylene receptor^25^ in WT (Fig. 6B). At 50 µM Cd, both *ddc* and WT exhibited *ETR2* and *ERF1* overexpression suggesting an upregulation of ethylene signalling (Fig. 6B); no differences occurred in *ddc vs* WT (Fig. 6B).

In summary, in control conditions GP related to ethylene biosynthesis was upregulated in *ddc vs* WT. Cd determined a downregulation and upregulation of this GP in *ddc* and WT, respectively. Concerning hormone signalling, at the highest Cd concentration in both *ddc* and WT an upregulation of this GP occurred.

### Salicylic acid

Regarding SA, only the GP related to signalling resulted differentially expressed. Under control conditions, the GP related to SA signalling showed similar EP in *ddc* and WT. However, *PR1*, a useful molecular marker for the systemic acquired resistance (SAR) in response to pathogens^26^, was downregulated in *ddc vs* WT (Fig. 6C). 25 μM Cd induced a downregulation of genes codifying TGA10 transcription factor only in the *ddc* (Fig. 6C**).** Whereas, 50 μM Cd induced a downregulation of *PRB1* in both *ddc* and WT and of *TGA8* only in *ddc* (Fig. 6C). In *ddc vs* WT, differences were found only at 25 µM Cd, with the downregulation of *TGA10* and *CAPE3* (Fig. 6C).

Altogether, these results evidenced a Cd-induced downregulation of this GP, likely resulting in an impairment of hormone signalling in both WT and *ddc*, but in the latter this effect already occurred at the lowest Cd concentration.

### Phytohormone level

Based on the major effects induced by 25 µM Cd treatment, hormone quantification was carried out on plants exposed to this concentration, compared to untreated control plants.

**Figure 7:**
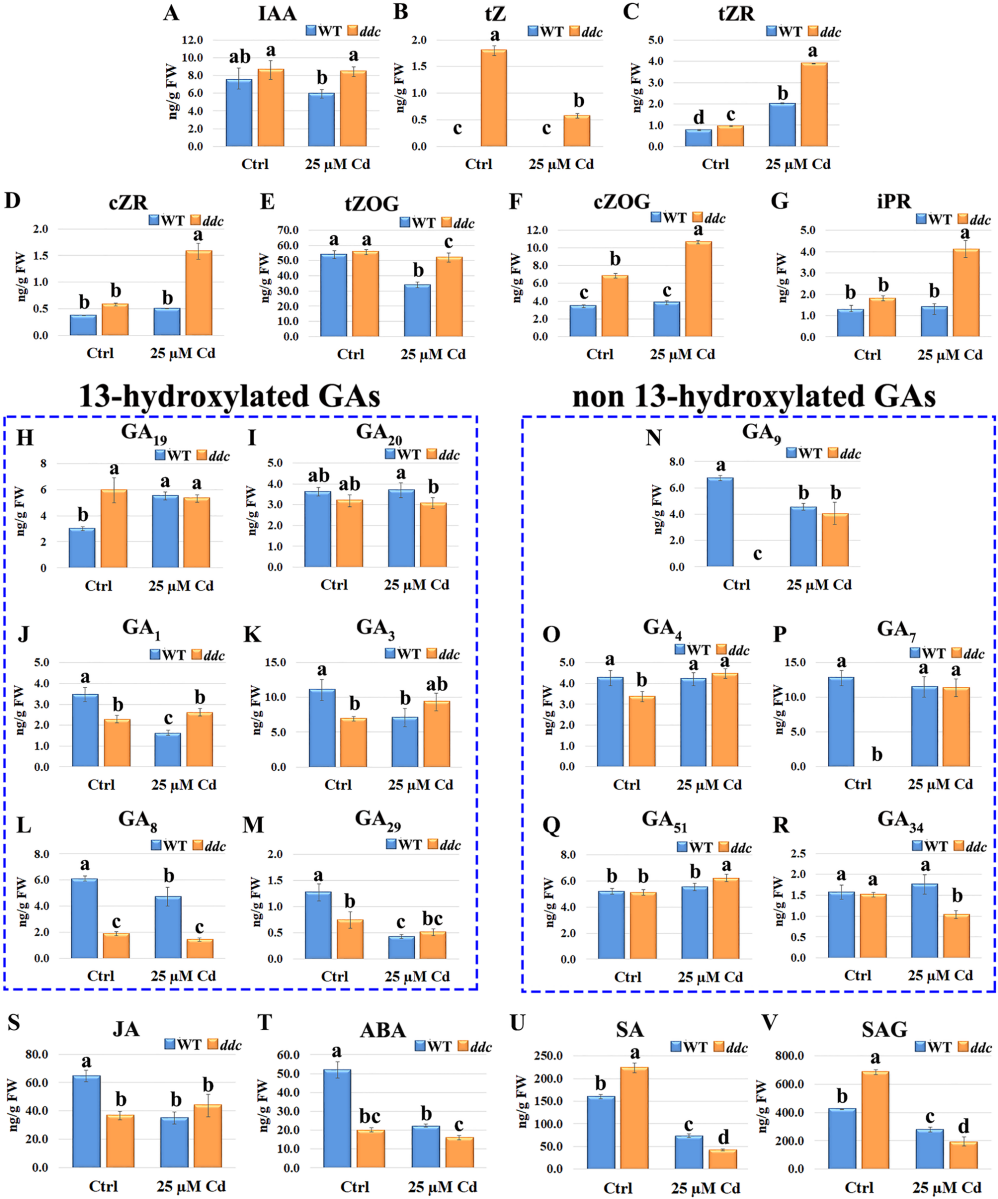
(**A**) IAA, (**B**–**G**) CKs, (**H**–**R**) GAs, (**S**) JA, (**T**) ABA, (**U**) SA and (**V**) SAG amount in *A. thaliana ddc* mutant and WT plants grown in Ctrl conditions and treated with 25 µM Cd estimated by GC–MS. The results represent the mean value (± SD) of three independent biological replicates. Statistical analysis was performed by using two-way ANOVA with Tukey post hoc test (*P* ≤ 0.05) after Shapiro–Wilk normality test. Means with the same letter are not significantly different at *P* ≤ 0.05.

Under control conditions, IAA amount was higher in *ddc* than WT, although not significantly. After Cd treatment, a decreasing trend was observed only in WT, resulting into a significant lower level as compared to *ddc* (Fig. 7A).

Concerning CKs, both biological active (*tZ*) and inactive conjugate (*tZR, cZR, tZOG, cZOG*, iPR) forms were analysed (Fig. 7B–G). Under control conditions, all analysed CKs were present in *ddc,* but CKs conjugate forms and above all *O*-glycosylated exhibited the highest levels (Fig. 7B–G). By contrast, in WT *tZ* was not detectable and all the other CKs forms exhibited a lower level compared to the mutant, which appeared significant for *tZR* and *cZOG* (Fig. 7B–G). Following Cd treatment, CKs levels increased in *ddc*, except for *tZ* decrease. In WT, the unique Cd effect dealt with *tZR* increase and *tZOG* decrease. Consequently, under Cd treatment the level of all CKs forms remained higher (from 0.25 to 3 times) in *ddc* than in WT (Fig. 7B–G).

Concerning GAs, precursors (GA_9_, GA_19_, GA_20_), biologically active forms (GA_1_, GA_3_, GA_4_, GA_7_) and catabolites (GA_8_, GA_34_, GA_29_, GA_51_) were analysed (Fig. 7H–R). Under control conditions, GA_19_ amount was significantly higher in *ddc vs* WT, while the amount of GA_20,_ the other *in serie* precursor of hydroxylated forms, was comparable between the samples (Fig. 7H,I). Following Cd treatment, GA_19_ amount significantly increased in WT while a slight downtrend occurred in *ddc*, leading to comparable values in the two samples. The same trend was observed, but at less extent, for GA_20_ (Fig. 7H,I). Consistently, under control conditions also the amount of the active hydroxylated forms GA_1_ and GA_3_ was higher in WT than in *ddc* (Fig. 7J,K). Following Cd treatment, a decrease of their amount was detected only in WT, globally leading to a higher level of these GAs in *ddc* mutant compared to the WT (Fig. 7J,K). In addition, in *ddc* mutant also the related catabolites GA_8_ and GA_29_ were globally lower than in WT, under both control conditions and Cd treatment (Fig. 7L,M).

Differences were observed also for GA_9_, precursor of non-hydroxylated GAs: under Cd treatment its amount decreased in the WT and was instead induced in *ddc* mutant, resulting in a quite comparable value between the two samples (Fig. 7N). Consistently, the amount of active non-hydroxylated forms, GA_4_ and GA_7_, increased under Cd treatment only in *ddc* mutant; also in this case, at the end of heavy metal treatment, comparable values were detected in *ddc* and WT (Fig. 7O,P) In agreement with these results, following Cd treatment, the amount of catabolites GA_51_ and GA_34_ did not change in the WT, whereas in *ddc* it increased and decreased, respectively (Fig. 7Q,R).

As evident in Fig. 7S–V, differences were reported also for JA, ABA, SA and its predominant inactive conjugate, SA 2-*O*-β-D-glucoside (SAG). Under control conditions both JA and ABA amount was significantly lower in *ddc vs* WT and significantly decreased following Cd treatment only in the WT (Fig. 7S,T). Notwithstanding, under such condition the ABA amount remained lower in *ddc* than in WT while JA values were comparable in the two samples due its light, but not significant, increase in *ddc* (Fig. 7S,T). By contrast, under control conditions both SA and SAG amounts were significantly higher in *ddc* than in WT (Fig. 7U,V). Following Cd treatment, their amounts significantly decreased more in *ddc* than in WT, leading to an opposite condition (Fig. 7U,V).

### Testing of the involvement of *SUPPRESSOR OF DRM1 DRM2 CMT3 (SDC)* gene in *ddc* response to Cd

Finally, we planned to inquire on the possible involvement of *SDC* gene in the response of *ddc* triple mutant to Cd exposure. Indeed, it has been reported that in *ddc* mutant the misexpression of such gene, which encodes a F-Box protein, is ultimately responsible of the developmental phenotypes of *ddc*, such as curled leaves and reduced growth, as evidenced by its reversion in the *drm1 drm2 cmt3 sdc* quadruple mutant^27^. Note that in the WT *SDC* is silenced, being methylated in all its sequence contexts because of the redundant action of DRM2 and CMT3 enzymes. By contrast, in *ddc*, where *DRM2* and *CMT3* expression is silenced, the loss of non-CG methylation in the promoter region of *SDC* F-box gene determines its overexpression^27^.

According to the above mentioned data^27^, we firstly verified that under control conditions *SDC* resulted silent in the WT and overexpressed in *ddc* also in our transcriptomic analysis (data not shown, complete raw transcriptomic data are available at NCBI SRA under the BioProject accessionPRJNA641242;https://www.ncbi.nlm.nih.gov/Traces/study/?acc=PRJNA641242). Moreover, our data also showed that at the transcriptomic level *SDC* is not modulated by Cd since its expression level did not significantly change in *ddc* nor in WT whatever heavy metal concentration was applied.

Thereafter, we tested the involvement of *SDC* in the growth response of *ddc* mutant under Cd exposure, by monitoring primary root length of *Arabidopsis thaliana* WT, *ddc* and *sdc* plants grown under the following conditions: (i) on growth medium without Cd as control (Ctrl) (ii) on a medium supplemented with 25/50 µM Cd; (iii) limited to the WT and *sdc* mutant plants, on a medium supplemented with 25/50 µM Cd plus 15 µM 5-Azacytidine (5-Aza), an inhibitor of DNA methylation applied in order to mimic the hypomethylated state of *ddc* mutant.

**Figure 8:**
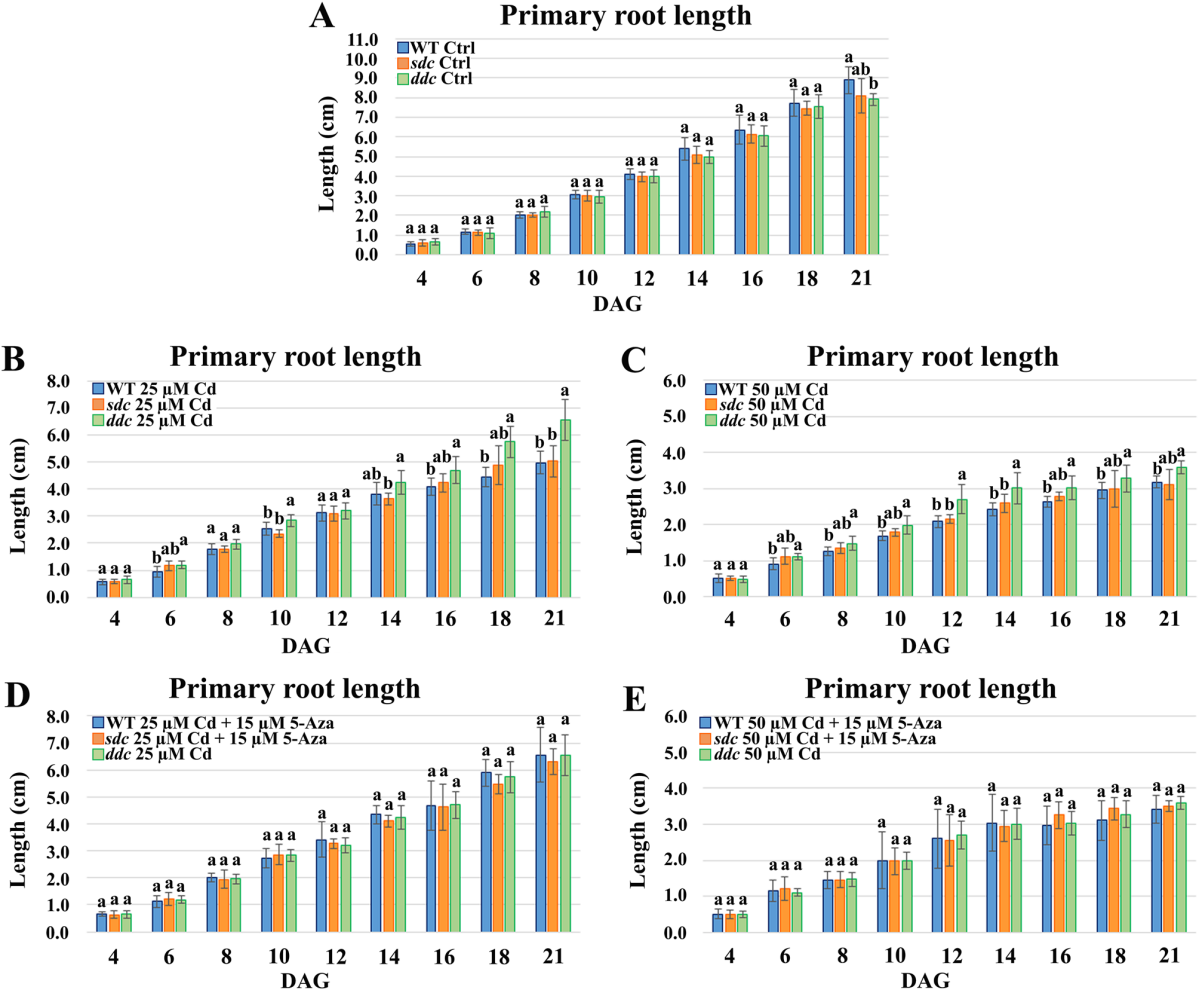
Primary root length of WT, *sdc* and *ddc* plants of *A. thaliana*, germinated and grown in long day condition (**A**) on growth medium without Cd as control (Ctrl), (**B**,**C**) on a medium supplemented with 25/50 µM Cd, (**D**,**E**) limited to WT and *sdc* plants, on a medium supplemented with 25/50 µM Cd plus 15 µM 5-Azacytidine (5-Aza). Root length was monitored up to 21 days after germination (DAG) every two days from germination. The results represent the mean value (± SD) of three independent biological replicates (n = 45). Statistical analysis was performed between samples at the same growth stage, by using two-way ANOVA with Tukey post hoc test (*P* ≤ 0.05) after Shapiro–Wilk normality test. Means with the same letter are not significantly different at *P* ≤ 0.05.

Under control conditions, all three samples showed a similar root length. However, at 21 DAG, root was lightly shorter in *ddc vs* WT, while *sdc* displayed an intermediate length (Fig. 8A). Under both Cd treatments, roots were averagely longer in *ddc* than in WT. Again, *sdc* roots exhibited an intermediate length, more similar to WT than *ddc* (Fig. 8 B,C). Interestingly, WT and *sdc* plants treated with Cd plus 5-Aza had longer roots than the plants treated only with Cd, and quite comparable to *ddc* roots exposed to Cd (Fig. 8 D,E).

## Discussion

This study showed that, under a long-lasting Cd treatment, *A. thaliana ddc* mutant exhibited a better growth performance than WT plants especially at 25 µM Cd concentration, tightly related to a differential modulation of GPs highly relevant for plant growth.

Attention was mainly focused on GPs related to hormones, key molecules in the control of plant growth and stress response, whose action is regulated by interconnected epigenetic mechanisms^12,28^. Accordingly, all hormone-related GPs were differentially modulated in *ddc vs* WT under control conditions but mainly under Cd stress, although at different extent for each hormone class. That is consistent with literature data showing that many plant hormones and other signal molecules are involved in Cd sensing and downstream plant response^10,29^. However, the general picture is somehow controversial differing in relation to species, plant organ and age, concentration and duration of metal exposure^30^.

In our work, for all hormone classes the major differences between *ddc* and WT occurred at the lowest Cd concentration (25 μM Cd). In particular, for auxin Cd impact was prominent on hormone conjugation rather than biosynthesis. Namely, the two GPs related to auxin biosynthesis were oppositely affected by Cd in both *ddc* and WT, likely as a compensatory mechanism to maintain hormone level. However, at 25 μM Cd IAA amount, which is the most relevant auxin, was higher in *ddc* than WT, evidencing that maintenance of bioactive auxin level was more efficient in the mutant. The downregulation of genes involved in auxin conjugation detected in *ddc* under such treatment provides a suitable explanation for this behaviour.

Transcriptomic data also suggested an enhancement of auxin signalling in *ddc vs* WT at 25 μM Cd. Notably, in *Arabidopsis* a Cd-induced simultaneous decrease of IAA content and increase of IAA oxidase activity resulted into a downregulation of numerous auxin-responsive and growth-related genes^31^. Moreover, under Cd exposure high level of IAA were found to prevent growth inhibition and increase heavy metal tolerance^32^. Therefore, although at post-transcriptional level a different regulation can occur, the better growth performance of *ddc* under 25 μM Cd could be warranted by transcriptomic changes addressed to prevent hormone inactivation and enhance its signalling. Interestingly, in our previous work an organ-specific and opposite alterations of auxin translocation and homeostasis was detected in *ddc* root and leaf as compared to WT, tightly related to mutant phenotypic alterations^33^. Furthermore, such hormone pathway alterations were associated to organ-specific changes in the expression level of auxin-related genes, which in some cases were in turn associated to gene-specific defective methylation level^33^. Altogether, these results highlight that a relationship between methylation status and auxin metabolism and translocation play a relevant role in modulating growth and development of *ddc* under both control conditions and Cd stress.

CKs also resulted differentially impacted by Cd in *ddc vs* WT both at transcriptomic and biochemical level. As for all hormones, CKs homeostasis relies on the balance between biosynthesis, catabolism and/or inactivation^34^. Transcriptomic major differences dealt with GPs related to CKs catabolism and inactivation rather than biosynthesis, at least at the lowest Cd concentration. Literature data showed an increase of CKs catabolic oxidation in *Triticum durum* exposed to 0.04 mM Cd^35^. A more complex picture was detected in our samples under a long-lasting treatment at a lower concentration (25 μM Cd). Namely, a Cd-induced downregulation of cytokinin-oxidase genes was observed only in *ddc.* Simultaneously, GPs related to CKs inactivation through *N*-glycosylation were upregulated in both *ddc* and WT, while *O-*glycosylation was upregulated only in *ddc*. Accordingly, hormone quantification showed that CKs *O*-glycosylated forms increased in Cd-treated *ddc* and were more abundant than in WT. Notably, while *N-*glycosylation causes irreversible CKs inactivation, *O-*glycosylated CKs represent the hormone fraction available for storage and transport, that can be reconverted in active CKs by β-glucosidases^16^. Therefore, the genetic modulation implemented by *ddc* under 25 µM Cd concentration was addressed to preserve CKs pool. That is consistent with the high CKs level detected in *ddc vs* WT at 25 μM Cd, despite the GP related to hormone biosynthesis exhibited a comparable EP. Incidentally, CKs are essential for counteracting leaf senescence, protecting photosystems and enhancing photosynthesis^36^. Accordingly, under Cd treatment leaf number and area was higher in *ddc vs* WT, evidencing that leaf growth was less impaired in *ddc*. Therefore, the genetic modulation underway in *ddc* could represent an important strategy to assure resistance to Cd stress.

On the other hand, also GAs play a relevant role in leaf development^37^. A downregulation of pathways related to the biosynthesis of active GAs occurred in *ddc* at 25 μM Cd. Likely as a compensatory defence mechanism, a strong downregulation of the GPs related to GAs inactivation and an enhancement of hormone signalling also occurred in *ddc* at the lowest Cd concentration.

A rather complex picture emerged for JA, SA and ABA, which represent the most relevant hormones for the perception and downstream response of plants to stresses, including heavy metals^38^. Namely, under control conditions, at transcriptomic level JA biosynthesis was downregulated and a lower hormone amount was detected in *ddc vs* WT. Whereas, for SA and ABA a post-transcriptional regulation can be envisaged, as already demonstrated for ABA^39^, since GPs related to their biosynthesis showed similar EP in *ddc vs* WT, despite of significantly higher and lower hormone level detected in *ddc,* respectively.

At 25 μM Cd, a downregulation of biosynthesis-related GPs and hormone level, was observed for all three hormones. However, for SA and ABA this effect was more pronounced in *ddc* than WT, while JA was more affected in WT than *ddc.* likely explaining the comparable JA level in Cd-treated *ddc* and WT. 25 μM Cd also induced a downregulation of GP related to ABA degradation only in *ddc vs* WT, probably as a compensatory mechanism for maintaining adequate hormone level. An opposite effect was observed for GP related to ABA signalling, which appeared upregulated in the WT and downregulated in *ddc* whatever concentration was used.

This scenario was unexpected, since it is largely documented that level and activity of these stress-related hormones, mainly of ABA, usually increase following abiotic stress, including heavy metal^40^. However, the exact mechanisms of hormone action and their crosstalk with the whole signalling network of plant under stress are yet to be fully clarified. In addition, hormone dynamic under stress largely depends on the species, the plant organ and growth stage, the stress intensity and duration^40^. For example, Cd treatment was found to induce a differential JA accumulation in several plant species, including *A. thaliana* which exhibited a biphasic model, with an early hormone accumulation, followed by cyclic decreases and increases^41^. Notably, low JA concentrations act as protectant against Cd stress, while higher concentrations induce toxic effects, such as ROS accumulation, root growth inhibition and lipid peroxidation^42^. Similar toxicity is displayed by high levels of SA, which has been proposed as the ‘life or death switch’ of cells^43^. Moreover, negative effects on plant growth can be exerted also by high levels of ABA, which acts as an antagonist of GAs action^39^. Therefore, it is likely that under the long-lasting Cd treatment that we applied, plant activity was directed to avoid toxic effects related to a prolonged activation of all these three hormone classes, by decreasing their level and/or downregulating their signalling. Interestingly, in *ddc* which exhibited the best growth performance, these adaptive responses were more pronounced.

In summary, our results clearly showed that, under a prolonged metal exposure and within a specific threshold concentration (i.e. 25 μM Cd), a differential transcriptional modulation of hormone pathways is a key mechanism for the capacity of *A. thaliana ddc* mutant, defective in methylation, to better counteract Cd toxicity compared to WT. Moreover, *ddc* prompt response at the lowest Cd concentration (i.e. 25 μM Cd) also evidenced that such aptitude was associated to its greater capacity to sense heavy metal stress and put in place an early modulation of gene expression. We propose that this behaviour is related to the higher genome plasticity conferred to *ddc* by DNA hypomethylated status.

In this context it is relevant to recall that the overexpression of the *SDC* F-box gene in *ddc*, due to the loss of non-CG methylation in its promoter region, is reported to be ultimately responsible of the developmental phenotypes of this mutant, such as curled leaves and reduced growth^27^.

In line with this evidence, also our transcriptomic analysis evidenced that, under control conditions, *SDC* is silent in the WT and overexpressed in *ddc*. Furthermore, since *SDC* expression level did not significantly change in *ddc* nor in WT whatever Cd concentration was applied, our data also evidenced that, at the transcriptomic level, *SDC* expression is not modulated by Cd. Moreover and very interestingly, the analysis of root growth allowed us to verify that under Cd treatment the roots of WT and *sdc* plants where shorter than *ddc* ones whereas, following treatment with both Cd and the DNA methylation inhibitor 5-Aza, both displayed longer roots, quite comparable to *ddc* ones. Thus, under Cd exposure a root length comparable to *ddc* was reported only in 5-Aza-treated plants mimicking *ddc* global DNA hypomethylation status, independently by the activation or the silencing of *SDC* which occurs in *ddc* and *sdc*, respectively. These results pointed out two important evidences: (i) a global DNA hypomethylation is certainly involved in a better plant response to Cd stress; (ii) such response is independent by *SDC* gene.

Therefore, it appears that *SDC* activity is not directly related to the response to Cd. Future analysis of methylome status of *ddc* and WT under Cd treatment could give further insight into the different and interacting loci with a direct and or indirect role in the transduction pathways and response mechanisms to Cd stress of *ddc* mutant.

**Figure 9:**
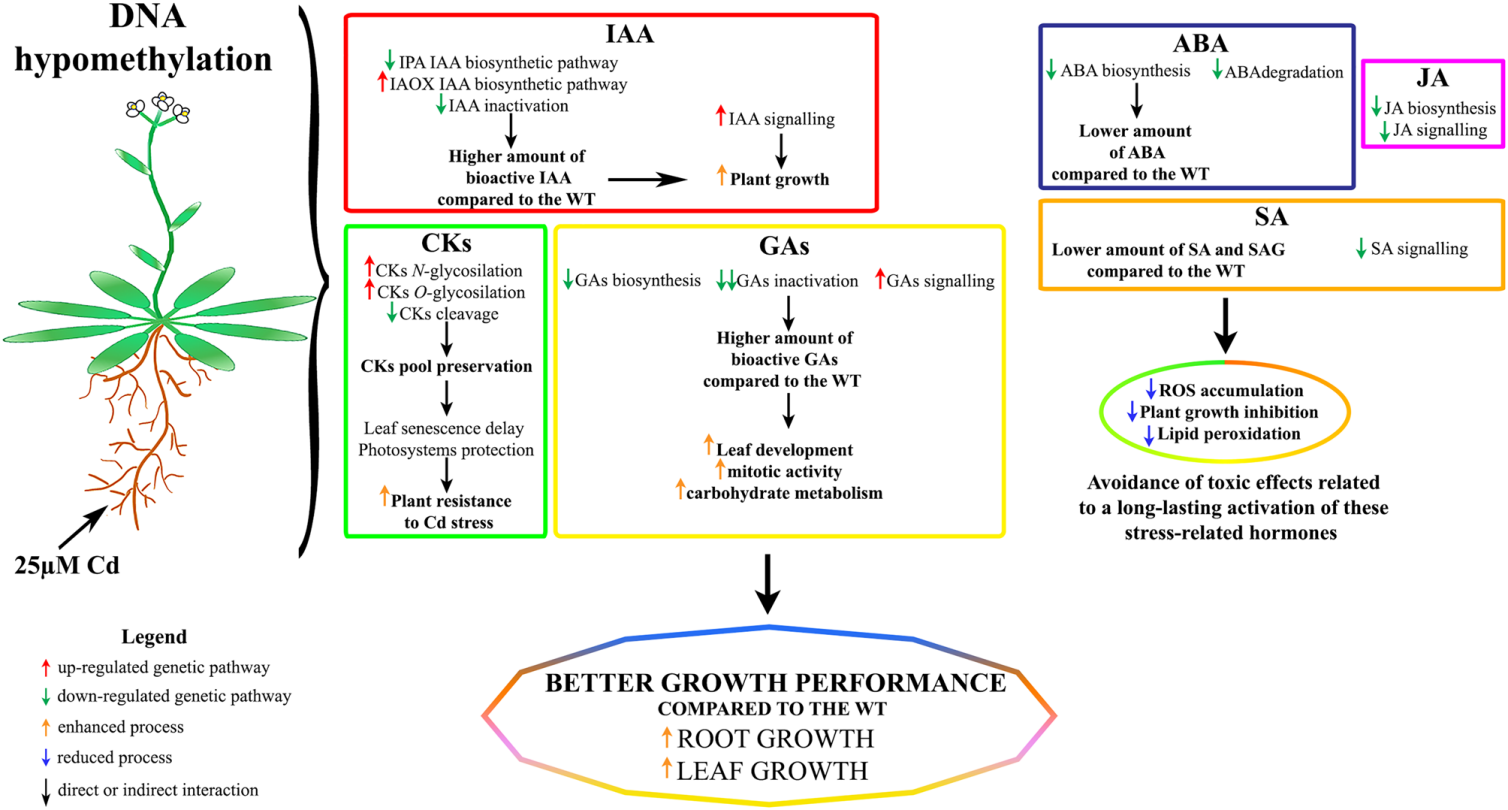
Scheme summarizing the modifications in hormone-related genetic pathways in *A. thaliana ddc* mutant plants under 25 µM Cd stress. The related effects of these modifications are also indicated.

At present, on the basis of our results, the following scheme is proposed to link transcriptomic and hormonal differences to the major Cd tolerance exhibited by *ddc* (Fig. 9). The emerging picture is that plant activity is directed to enhance and/or maintain the level and signalling of hormones which are relevant in sustaining the growth, such as auxins, CKs and GAs more than those of hormones specifically related to stress response such as JA, ABA and SA. This could represent the plant strategy, more effective in *ddc* than in WT, to avoid the negative effects of long-lasting activity of stress-related hormones. In view of the emerging relationship between the phytohormone action and epigenetic mechanisms^12^, this supposed role of methylation status in modulating plant strategy for ‘life or death switch’ under stress condition appears relevant at both theorical and applicative level.

## Methods

### Plant Lines and growth conditions

Plants of *Arabidopsis thaliana* (L.) Heynh ecotype Columbia-0 (Col-0), *drm1 drm2 cmt3.11 (ddc*) DNA methylation mutant and *suppressor of drm1 drm2 cmt3* (*sdc*) silencing mutant, both in Col-0 background, were used. *Arabidopsis thaliana* seeds and plants were handled according to^45^ and by following the methods recommended by Arabidopsis Biological Resource Center (ABRC; https://abrc.osu.edu/) available at https://www.arabidopsis.org/download_files/Protocols/abrc_plant_growth.pdf.

Seeds were surface sterilized and sown on Petri dishes containing half-strength MS medium^45^. Regarding the different treatments, medium was supplemented with 25 µM and 50 μM Cd, as described by^10^, and/or 15 µM 5-Azacytidine (5-Aza), according to^46^. Detailed information on plant lines and selected treatment conditions are reported in Supplementary Methods S1 online. We will refer to the different samples as follows: WT Ctrl, WT 25 µM Cd, WT 50 µM Cd, WT 25 µM Cd + 15 µM 5-Aza, WT 50 µM Cd + 15 µM 5-Aza, *ddc* Ctrl, *ddc* 25 µM Cd, *ddc* 50 µM Cd, *sdc* Ctrl, *sdc* 25 µM Cd, *sdc* 50 µM Cd, *sdc* 25 µM Cd + 15 µM 5-Aza, *sdc* 50 µM Cd + 15 µM 5-Aza.

### Cd quantification

Cd quantification was performed according to^47^ on samples collected at 21 DAG. Three independent replicates were carried out. For statistical analysis, two-way ANOVA with Tukey post hoc test (*P* ≤ 0.05) was applied after Shapiro–Wilk normality test. The results of such analysis are provided in Supplementary Fig. S10 online.

### Growth parameters analysis

Root length was monitored in plants grown in vitro in a vertical position every two days until 21 DAG. Rosette leaf number and area were evaluated in plants grown in round Petri dishes for 21 DAG. Leaf series was obtained as described by^48^. Measurements were performed by scanning the plates and analysing the resulting images by using ImageJ software (https://imagej.nih.gov/ij/). Three independent replicates were performed (n = 45). Statistical analysis was carried out by using Student’s *t*-test (*, *P* ≤ 0.05; **, *P* ≤ 0.01; ***, *P* ≤ 0.001) between *ddc vs* WT grown in the same conditions.

### Total RNA extraction

Total RNA was isolated from 100 mg of plant tissue by using the RNeasy Plant Mini kit (Qiagen, Hilden, Germany) and DNA contamination was eliminated through on column DNase digestion (RQ1 RNase-Free Dnase, Cat. Nr. M6101). Extracted RNA was quantified by using the Qubit RNA BR (Broad-Range) Assay Kit, while its integrity was checked by using an Agilent 2100 Bioanalyzer (Agilent Technologies). Only RNA samples with an RNA integrity number ≥ 8 were subsequently used.

### RNA-seq

cDNA libraries were constructed from 1 µg of total RNA, using the Illumina TruSeq Stranded Total RNA Sample Preparation Kit (Illumina, San Diego, CA, USA). Quality of the obtained libraries and the fragments length were verified on the Bioanalyzer 2100 by using an Agilent 2100 DNA 1000 Kit and quantified by fluorimetry using the Qubit dsDNA HS (High sensitivity) Assay kit (Q232854). The sequencing of the cDNA libraries was carried out on Illumina Genome Analyzer IIx (SCS v2.10) platform.

### Preprocessing and analysis of RNA-seq data

RNA-Seq reads in FASTQ format were inspected using FASTQC program (http://www.bioinformatics.babraham.ac.uk/projects/fastqc/). Only reads with Phred quality score Q > 30 (Q30 Quality Score) were used (from 90 to 95%, for a total of 29.4 Giga reads of 50 bp paired-end reads). Reads were cleaned by using Trim Galore (http://www.bioinformatics.babraham.ac.uk/projects/trim_galore/) and matched to Arabidopsis TAIR10 gene sequences database, allowing for two mismatched bases. Only the alignments unique and concordant in SAM format were converted in binary BAM format by SAMtools. Basic statistics were calculated using Picard tools (CollectRnaSeq Metrics.jar) (http://picard.sourceforge.net/). Transcriptome quantification and RNA differential expression were performed using CuffDiff2 (http://cufflinks.cbcb.umd.edu/) software version 2.1.1, as described by^49^.

Gene expression levels were determined by FPKM calculation, using the Cufflinks method^50^^.^ Bioinformatic analysis was performed by multiple pairwise comparisons of gene expression levels. Differentially expressed genes (DEGs) were selected on the basis of fold change (FC) (5 ≥|log_2_ FC|≥ 2 and FDR < 0.05).

### Gene enrichment analysis

A functional annotation analysis of DEGs was performed by using Gene Ontology (GO) annotations^51^ (http://www.geneontology.org/). Gene Enrichment analysis, based on biological process ontology and KEGG database, was performed by selecting the over-represented GO terms through the ClueGO plug-in of the Cytoscape software^52,53^. The significance of each term and group was determined by the calculation of a Bonferroni-corrected *P*-value using the hypergeometric distribution. Only the GO terms with *P* ≤ 0.05 were selected.

### Analysis of hormone-related pathways

The analysis of hormone-related pathways was performed by using the KEGG Mapper tool^54^ (https://www.genome.jp/kegg/mapper.html) for hormones signalling pathways and the online tool PlantMetGenMAP^55^ (http://bioinfo.bti.cornell.edu/cgibin/MetGenMAP/home.cgi), which allows a large-scale exploration of gene expression data-set and the identification of the significantly altered biochemical pathways and biological processes through robust statistical tests.

### Libraries results validation through quantitative Real-Time PCR (qRT-PCR)

Transcriptomic analysis was validated by estimating the expression level of 14 hormone-related key genes. Primers sequences and additional information regarding the selected genes are shown in Supplementary Table S1 online. Detailed information is reported on Supplementary Methods S1 online.

### Hormone level quantification

Hormone level quantification was performed on WT and *ddc* mutant plants grown in control conditions and under 25 µM Cd treatment, collected at 21 DAG. Detailed information on hormone level quantification methods is reported on Supplementary Methods S1 online.

## Supplementary Information

Supplementary Information.

## Data Availability

Raw transcriptomic data generated during and/or analysed during the current study are available at NCBI SRA under the BioProject accession PRJNA641242 (https://www.ncbi.nlm.nih.gov/Traces/study/?acc=PRJNA641242).
