# Supplementary material for: In Arabidopsis thaliana Cd differentially impacts on hormone genetic pathways in the methylation defective ddc mutant compared to wild type
Source: Sci Rep. 2021 May 26;11:10965. doi: 10.1038/s41598-021-90528-5 (PMC8154917; doi:10.1038/s41598-021-90528-5)
Supplement: Supplementary file 1 — Supplementary Information. [file 41598_2021_90528_MOESM1_ESM.pdf]

Supplementary material

Title: **In *Arabidopsis thaliana* Cd differentially impacts on hormone genetic pathways in the methylation defective *ddc* mutant compared to wild type**

Marianna Pacenza[1], Antonella Muto[1], Adriana Chiappetta[1], Lorenzo Mariotti[2], Emanuela Talarico[1], Piero Picciarelli[2], Ernesto Picardi[3], *Leonardo Bruno[1] and Maria Beatrice Bitonti[1]

[1]Department of Biology, Ecology and Earth Science of University of Calabria, Arcavacata di Rende, CS, Italy;
[2]Department of Agriculture, Food and Environment, University of Pisa, Pisa, PI, Italy;
[3]Department of Biosciences, Biotechnology and Biopharmaceutics, University of Bari, Bari, BA, Italy.

Authors emails:

Marianna Pacenza: mariannapacenza@gmail.com;
Antonella Muto: antonella.muto@unical.it;
Adriana Chiappetta: adriana.chiappetta@unical.it;
Lorenzo Mariotti: lorenzo.mariotti@unipi.it;
Emanuela Talarico: emanuela.talarico@unical.it:
Piero Picciarelli: piero.picciarelli@unipi.it;
Ernesto Picardi: ernesto.picardi@uniba.it;
* Leonardo Bruno, leonardo.bruno@unical.it, +39 0984 492963.
Maria Beatrice Bitonti: maria _beatrice.bitonti@ unical.it; b.bitonti@virgilio.it;

Methods S1

Plant Lines and growth conditions

Plants of *Arabidopsis thaliana* (L.) Heynh ecotype Columbia-0 (Col-0), *drm1 drm2 cmt3.11 (ddc*) DNA methylation mutant and *suppressor of drm1 drm2 cmt3* (*sdc*) silencing mutant, both in Col-0 background, were used. *ddc* triple mutant combines *drm1* (SALK_021316; AT5G15380) with T-DNA insertion in the sixth exon, *drm2* (SALK_150863; AT5G14620) with T-DNA insertion in the last exon, and *cmt3.11* (SALK_148381; AT1G69770) with T-DNA insertion in the eighth intron[1]. In *sdc* homozygous mutant the *SDC* (AT2G17690) insertion is Salk T-DNA_017593[2]. Seeds were purchased from the Nottingham *Arabidopsis* Stock Centre (NASC, http://arabidopsis.info/). Selection of homozygous mutants was conducted following the NASC standard protocol (http://signal.salk.edu/tdnaprimers.2.html).

Seeds were surface sterilized and sown one part in round and one part in square Petri dishes containing half-strength MS medium 1% sucrose and stratified at 4ºC for 48 h. The plated seeds were then exposed at 21ºC under long day condition (photoperiod, 16 h : 8 h, light : dark) with white light (neon fluorescent tubes "Radium NL Spectralux, cool white", 100 µmol m^{-2} s^{-1}) and 50% relative humidity until 21 days after germination (DAG) and used as control conditions (Ctrl). Square Petri dishes were maintained in vertical position for all the growth period.

For Cd treatment, medium was supplemented with 25 µM and 50 µM Cd by directly adding an aliquot of $CdCl_2$ water stock solution (100 mM) to medium immediately before placing it in the Petri dishes, as described by [3].

In order to mimic the global hypomethylated state of the *ddc* mutant, WT and *sdc* mutant were treated with the nonmethylable cytosine analog 5-azacytidine (5-Aza). Preliminary tests were performed with multiple 5-Aza concentrations and root length was monitored. 15 µM was selected as the treatment where WT root length was quite similar to the *ddc* grown in control conditions (data not shown). Subsequently, 5-Aza treatment was performed by the addition of an aliquot of a water stock solution of 0.1 M 5-Azacytidine (Sigma) to medium immediately before placing it in the Petri dishes, according to [4].

Regarding the combined 25/50 µM Cd plus 15 µM 5-Aza treatment, aliquots of $CdCl_2$ water stock solution (100 mM) and 5-Azacytidine water stock solution (0.1 M) were both added to the medium immediately before placing it in the Petri dishes, as described by [3,4].

Libraries results validation through quantitative Real-Time PCR (qRT-PCR)

Transcriptomic analysis was validated by estimating through qRT-PCR the expression level of 14 hormone-related key genes. To this aim, first-strand cDNA synthesis was performed by

SuperScript™ III Invitrogen USA, according to the manufacturer's instructions. Primers used for qRT-PCR analysis were designed using Primer-BLAST tool (https://www.ncbi.nlm.nih.gov/tools/primer-blast/)[5], with the exception of the housekeeping gene primers, that were designed by [6]. Primers pairs where selected on the basis of their robustness, specificity and consistency. Only the ones with an average efficiency between 0.95 and 1.0 were used. The housekeeping gene *AT2G28390* (*MONENSIN SENSITIVITY1, SAND*) was selected for normalization from independent trials of several housekeeping genes as the one that produced the most reproducible results across various cDNAs, as also described by [6]. The primer sequences are reported in Supporting Information Table S1.

Quantitative real-time PCR (qRT-PCR) was performed using STEP ONE instrument (Applied Biosystems). The results were analysed using STEP One Software 2.0 (Applied Biosystems), by using the ΔΔCt method[7]. As RNA-sequencing and qRT-PCR produce relative gene expression measures, one of the samples (WT grown in Ctrl condition) was chosen as reference, to which all the others were compared to obtain the $\log_2$ (FC) values used to evaluate concordance in gene expression by Pearson correlation coefficient calculation.

Statistical analysis was performed by using two-way ANOVA with Tukey post hoc test ($P \leq 0.05$) after Shapiro–Wilk normality test. Means with the same letter are not significantly different at $P \leq 0.05$.

Hormone level quantification

Plant material was ground in a mortar with 80 % MeOH (1:5 w/v). [13C6]-IAA (Cambridge Isotopes Laboratories Inc., Andover, MA), [2H6]-ABA (OlChemlm Ltd., Olomouc, Czech Republic), [2H4]-SA (CDN Isotopes Inc., Quebec, Canada) and [2H5]-JA (CDN Isotopes Inc.), labelled CKs [2H5]-tZ, [2H5]-tZR, [2H5]-cZR, [2H5]-tZOG, [2H5]-cZOG, [2H6] -iPR (OlChemlm Ltd., Olomouc, Czech Republic) and deuterated GAs ([17,17- 2H2]-GA9, [17,17- 2H2]-GA4, [17,17- 2H2]-GA34, [17,17- 2H2]-GA7, [17,17- 2H2]-GA51, [17,17- 2H2]-GA19, [17,17- 2H2]-GA20, [17,17- 2H2]-GA29, [17,17- 2H2]-GA1, [17,17- 2H2]-GA8, [17,17- 2H2]-GA3, [17,17- 2H2]-GA5 (obtained from Dr. L. N. Mander, Australian National University, Canberra, Australia), 50 ng each, were added as internal standards. Methanolic extracts were centrifuged (4000 g; 5 min), surnatants were collected and pellets were eluted with 80 % MeOH. The extraction was repeated three times. After adjusting the pH at 2.8, the methanol was evaporated under vacuum at 35 °C and the aqueous phase was partitioned with ethyl acetate (1:1 v/v). For GAs purification, the extracts were also dried and suspended in 0.3-0.5 ml of distilled water with 0.01% acetic acid and 10% methanol[8,9].

JA, SA, ABA, GAs and IAA were separated by reversed phase HPLC by using a Kontron instrument (Kontron Instruments, Munich, Germany) equipped with a variable wavelength UV detector SpectroMonitor 3100 (Milton Roy, Florida, USA) operating at 214 nm at a flow rate=1 ml min^{-1}. Samples were applied to a 150 mm×4.6 mm i.d. column, packed with Hypersil C18 particle size 5 μm (Thermo Fisher Scientific Inc., Waltham, MA, USA). Each fraction was dried in a rotary evaporator and resolved in MeOH for GC-MS analysis[8,9].

Quantitative determination of IAA, ABA, SA, GA and JA was performed by using a Saturn 2200 quadrupole ion trap mass spectrometer coupled to a CP-3800 gas chromatograph (Varian Analytical Instruments, WalnutCreek, CA, USA) equipped with a Mega 1MS capillary column (30 m×0.25 mm i.d., 0.25 m film thickness) (Mega, Milano, Italy).

After drying, samples were trimethylsilylated with 10µl of N,O-bis(trimethylsilyl) trifluoroacetamide (BSTFA) containing 1 % trimethylchlorosilane (TMCS) (Pierce, Rockford, IL, USA) at 70 °C for 1 hour and finally analysed by GC-MS. Plant hormones were identified by comparing full mass spectra with standard compounds. The concentration of each plant hormone in the extracts was determined from the peak area ratio of labelled and non-labelled ions of internal standard and endogenous hormone, respectively[8,9]. Final data were means of three biological replicates.

CKs quantification was performed by the laboratory of the National Research Council Canada on a UPLC/ESI-MS/MS utilizing a Waters ACQUITY UPLC system, equipped with a binary solvent delivery manager and a sample manager coupled to a Waters Micromass Quattro Premier XE quadrupole tandem mass spectrometer via a Z-spray interface. This analysis was carried on by using a modified procedure described in [10] and took on the Multiple Reaction Monitoring (MRM) function of the MassLynx v4.1 (Waters Inc) control software. The resulting chromatographic traces are quantified off-line by the QuanLynx v4.1 software (Waters Inc) wherein each trace is integrated and the resulting ratio of signals (non-deuterated/internal standard) is compared with a previously constructed calibration curve to yield the amount of analyte present (ng per sample). Calibration curves were generated from the MRM signals obtained from standard solutions based on the ratio of the chromatographic peak area for each analyte to that of the corresponding internal standard. The QC samples, internal standard blanks and solvent blanks were also prepared and analysed along each batch of tissue samples.

Supplementary Table S1. Primers used in qRT-PCR for libraries results validation. *Housekeeping gene, selected for normalization as the one that produced the most reproducible results across various cDNAs, as described by [6]. Gene description was obtained from the freely accessible database STRING (Search Tool for the Retrieval of Interacting Genes/ Proteins, https://string-db.org)[11].

TAIR Accession number	GENE NAME	Primers	Amplicon size (bp)
AT4G32540	*YUCCA 1; YUC1*	FW: 5'-TGGTCTTGCCACTTCAGCAT-3'<br>BW: 5'-GCGTAGGACTCAAGGTAGGC-3'	219
AT3G62980	*TRANSPORT INHIBITOR RESPONSE 1; TIR1*	FW: 5'-CTACGCGAGCTGAGAGTGTT-3'<br>BW: 5'-GGAAGCGAGTCATGTTGGGA-3'	190
AT2G01830	*WOODEN LEG; AHK4*	FW: 5'-GCTGCAGGAGCTCTCAAGAA-3'<br>BW: 5'-AGGTCGCGTGTATCACATCC-3'	247
AT1G79460	*GA REQUIRING 2; GA2*	FW: 5'-CTCGCGTTAAAGAAGTGGGG-3'<br>BW: 5'-AGCCCAATGGAATCGTCAGA-3'	178
AT1G14920	*GIBBERELLIC ACID INSENSITIVE GAI; DELLA* family gene	FW: 5'-ACGGTAACGGCATGGATGAG-3'<br>BW: 5'-CGACGGAGGATTAAGGTCGG-3'	218
AT1G20510	*OPC-8-0 CoA LIGASE1; OPCL1*	FW: 5'-ATTCCCCGTCGTTTGTCTCT-3'<br>BW: 5'-CACGATCGGGAGCTTCTTTG-3'	178
AT1G19180	*JASMONATE-ZIM-DOMAIN PROTEIN 1; JAZ1*	FW: 5'-CCTGATGTCAATGGAACTTTAGGC-3'<br>BW: 5'-TGGTGCAGTTTGAGACTCTGG-3'	228
AT3G14440	*NINE-CIS-EPOXYCAROTENOID DIOXYGENASE 3; NCED3*	FW: 5'-CGGTTTCTGGGAGATGGCTT-3'<br>BW: 5'-GGCTTAACAACAATGGCGGG-3'	202
AT2G27150	*ABSCISIC-ALDEHYDE OXIDASE 3; AAO3*	FW: 5'-TGGTTGCTTATGGTCTCGGT-3'<br>BW: 5'-AACACAGCAAAGCCTAACGG-3'	164
AT2G19590	*ACC OXIDASE 1; ACO1*	FW: 5'-ACGTTTTACAATCCGGCTGG-3'<br>BW: 5'-GCTGAATCCGCATTTCCCAT-3'	202
AT3G20770	*ETHYLENE-INSENSITIVE3; EIN3*	FW: 5'-GCTTACCGTATGGAGCAGCA-3'<br>BW: 5'-TGGACTGTGGGTTGAAGCAG-3'	244
AT4G39400	*BRASSINOSTEROID INSENSITIVE 1; BRI1*	FW: 5'-GCACGCAAAACTGCGGATTA-3'<br>BW: 5'-TATCCCTGACCCGGCTTGTA-3'	187
AT1G64280	*NONEXPRESSER OF PR GENES 1; NPR1*	FW: 5'-TTGTTTATCTGGCCGCCGAA-3'<br>BW: 5'-TTCTCGCTGACAAAACGCAC-3'	177

AT5G13320	*AVRPPHB SUSCEPTIBLE 3; PBS3*	FW: 5'-GAGGTTGTGAGGACGGGTTC-3'<br>BW: 5'-GTGGCCCTCCAAGAACCAAA-3'	169
AT2G28390	**MONENSIN SENSITIVITY1*; *SAND* family gene	FW: 5’-AACTCTATGCAGCATTTGATCCACT-3’<br>BW: 5’-TGATTGCATATCTTTATCGCCATC-3’	117

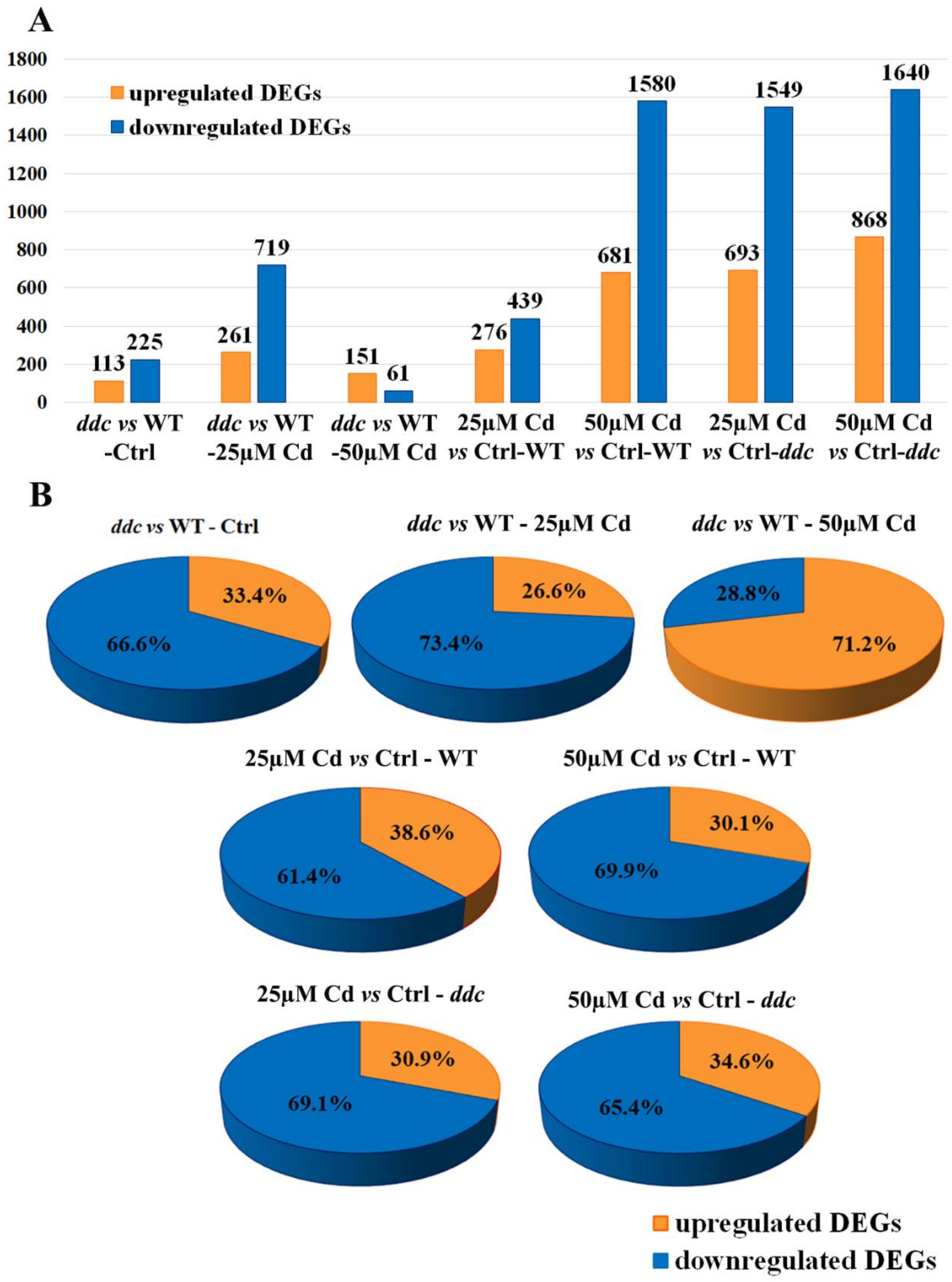

Fig. S1. A) Number and B) percentage of up and down-regulated DEGs in all the multiple pairwise comparisons of gene expression levels. Under control (Ctrl) conditions, relatively few genes were differentially expressed in *ddc* mutant as compared to the WT (338 DEGs). Following Cd exposure, a differential and dose-related increase of DEGs number was detected in *ddc* mutant and WT as compared to untreated plants (Ctrl). Indeed, the number of DEGs in 25 µM Cd *vs* Ctrl-*ddc* (2242 DEGs) was higher than the number of DEGs observed in 25 µM Cd *vs* Ctrl-WT (715 DEGs). Moreover, for each sample, (i.e. *ddc* mutant and WT) the highest number of DEGs (2261 and 2508 for WT and *ddc*, respectively) was found in the comparisons between the plants treated with the highest Cd concentration *vs* plants grown in Ctrl condition. Globally, from 61.4% to 69.9% of these DEGs were downregulated. In particular, under Ctrl condition 66.6% of the genes resulted downregulated in *ddc* mutant *vs* WT. Cd exposition induced a widespread down-regulation of gene expression in both *ddc* and WT plants. Therefore, a high percentage of down-regulated DEGs (73.4%) was also found in the comparison *ddc vs* WT-25 µM Cd. Interestingly, in the comparison *ddc vs* WT-50 µM Cd the majority of the DEGs resulted up-regulated (71.2%). In summary, these observations suggested that, under Cd stress, *ddc* mutant underwent to a differential modulation of gene expression compared to the WT.

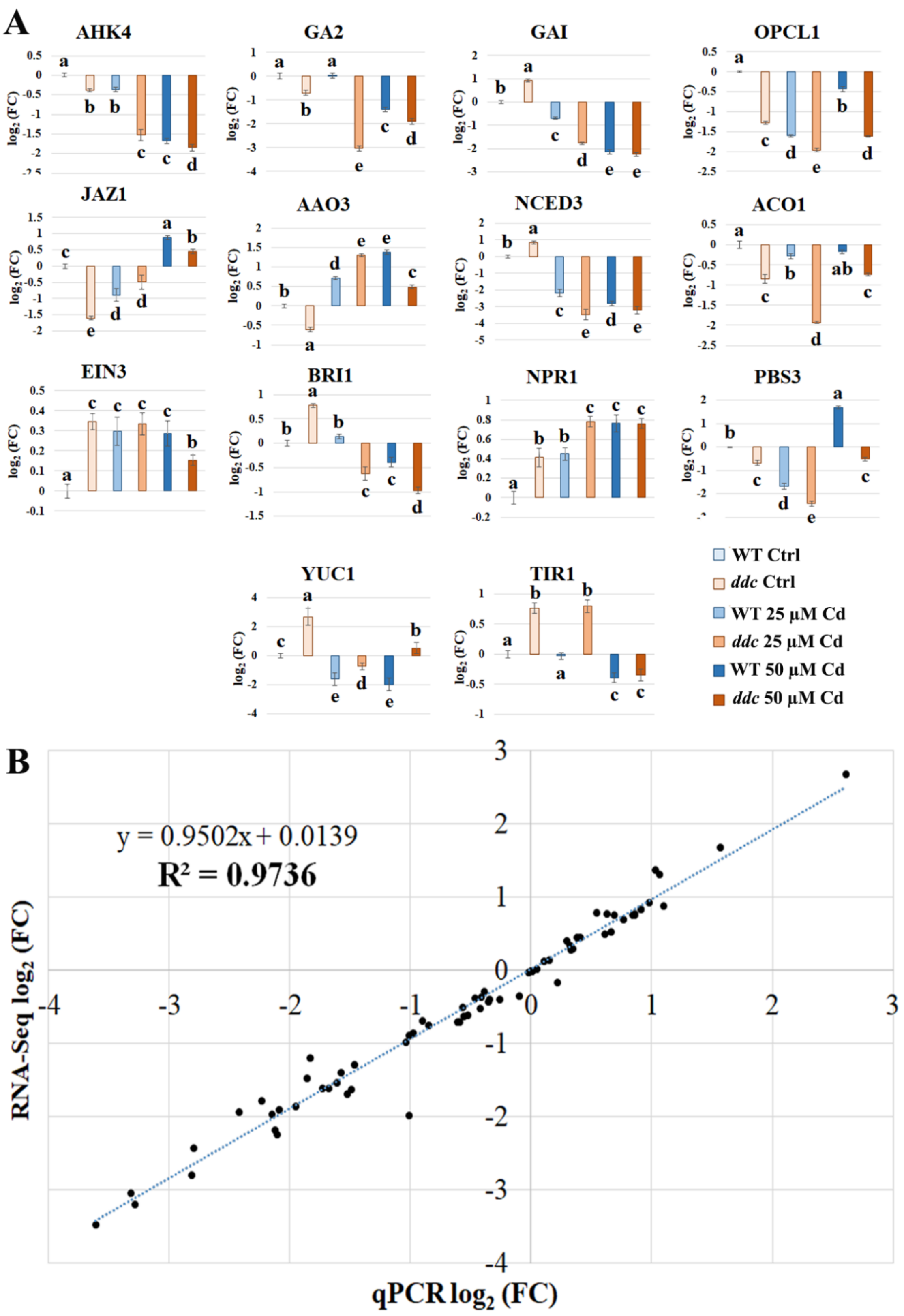

Fig. S2. Relative expression level by qRT-PCR of A) 14 selected DEGs involved in hormones metabolism and signalling. Data present the mean ± SE of three independent experiments. Statistical analysis was performed by using two-way ANOVA with Tukey post hoc test ($P \leq 0.05$) after Shapiro–Wilk normality test. Means with the same letter are not significantly different at $P \leq 0.05$; B) Linear correlation between log_2 (FC) values computed on RNA-Seq data and log_2 (FC) values detected by qRT-PCR for 14 selected genes. The estimates of fold change in expression level were highly consistent with those from RNA-Seq. $R^2 = 0.9736$.

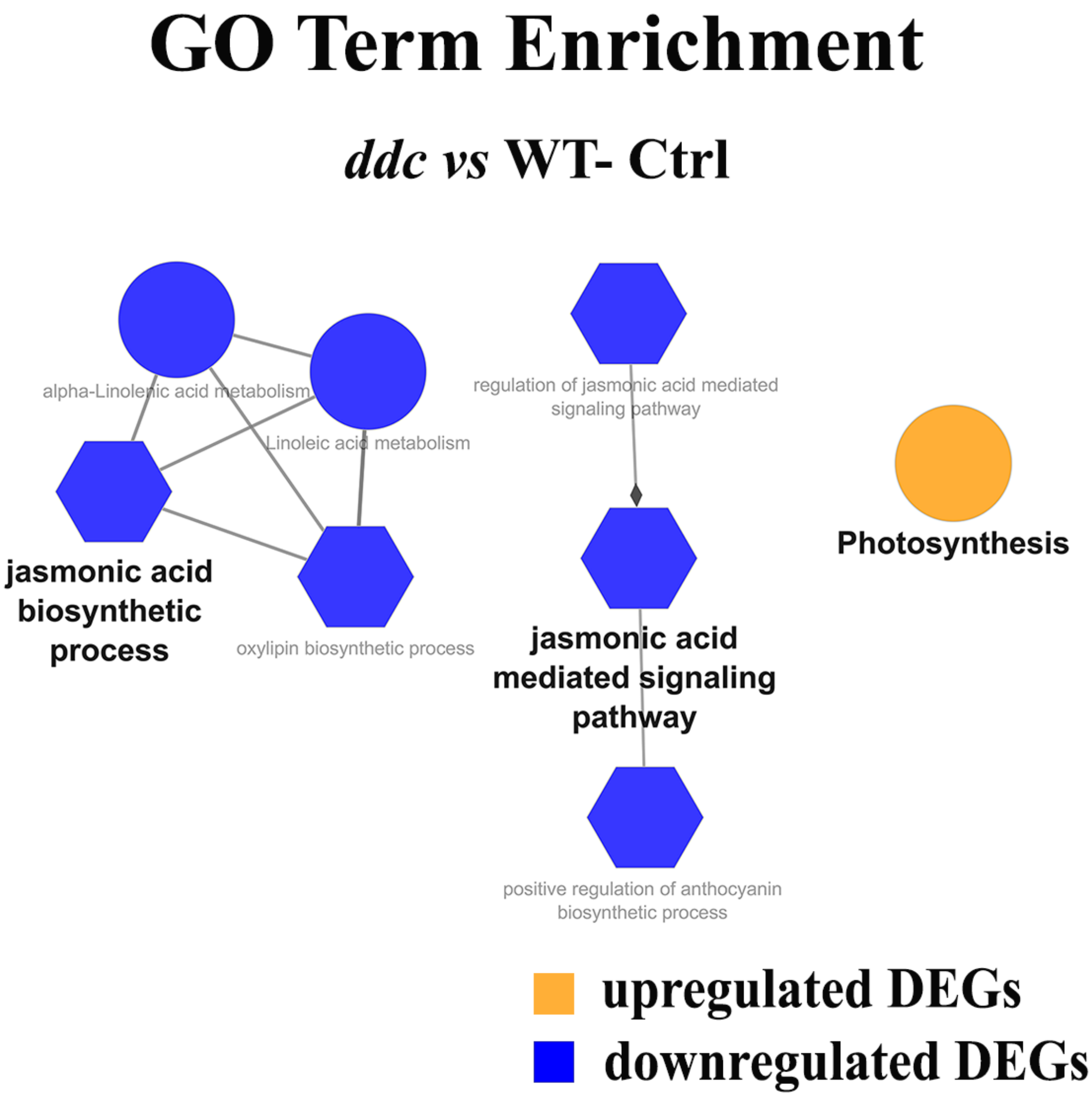

Fig. S3. Gene Enrichment analysis of the DEGs found in the comparison *ddc vs* WT-Ctrl. The analysis was performed by using ClueGO plugin of Cytoscape software[12] and the enriched genes were selected on the basis of the calculation of Bonferroni-corrected P-value by using the hypergeometric distribution. The upregulated genes are shown in orange, while the downregulated ones are shown in blue. The size of the nodes reflects the enrichment significance of the terms. Under control (Ctrl) conditions, the genetic pathways related to JA biosynthesis and signalling were significantly downregulated, and an upregulation of genetic pathways related to photosynthetic process was found in the *ddc* mutant compared to the WT.

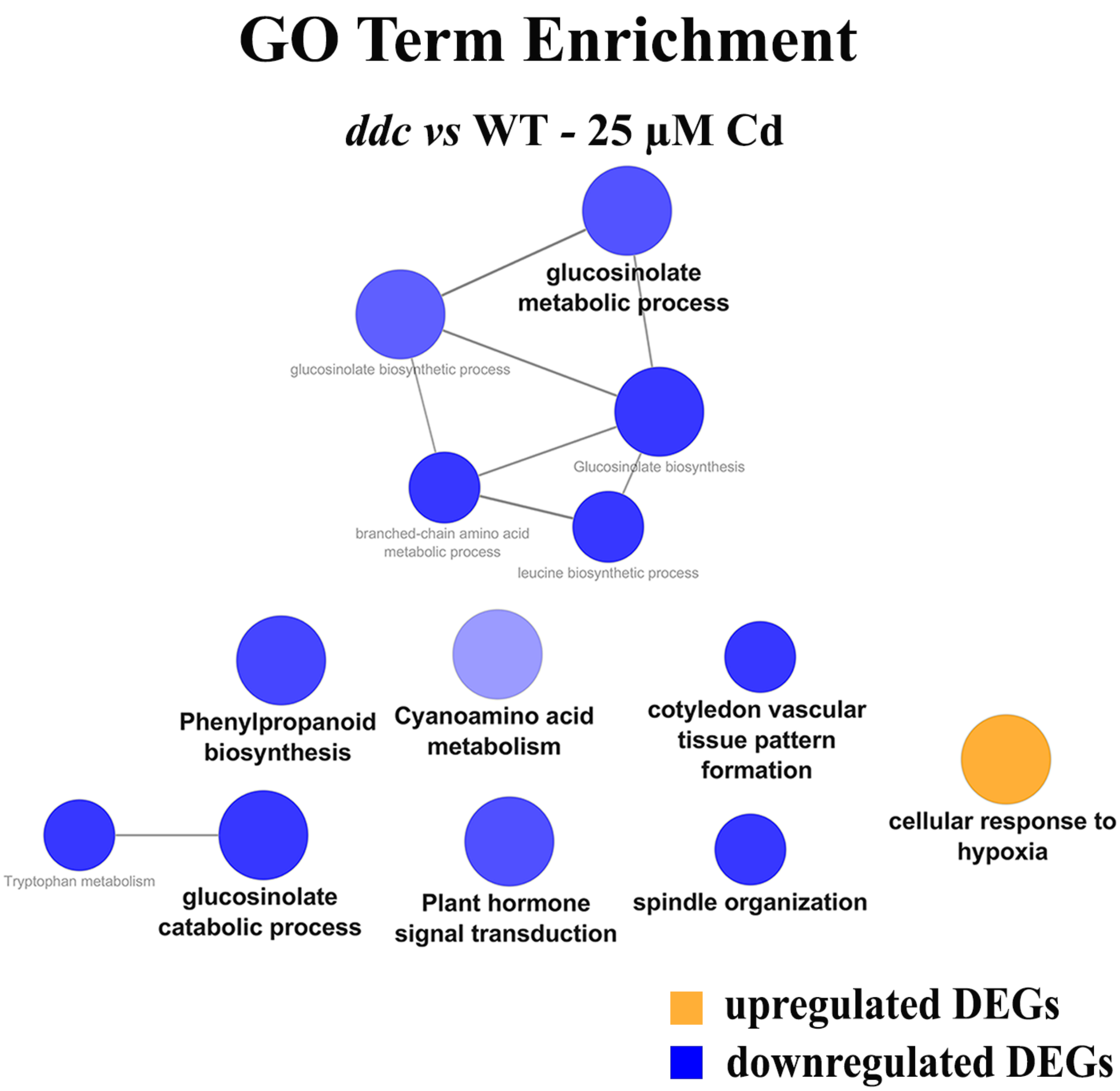

Fig. S4. Gene Enrichment analysis of the DEGs found in the comparison *ddc vs* WT-25 µM Cd. The analysis was performed by using ClueGO plugin of Cytoscape software[12] and the enriched genes were selected on the basis of the calculation of Bonferroni-corrected P-value by using the hypergeometric distribution. The upregulated genes are shown in orange, while the downregulated ones are shown in blue. The size of the nodes reflects the enrichment significance of the terms. Under 25 µM Cd treatment, the genetic pathway related to glucosinolate and cyanoamino acid metabolism, phenylpropanoid biosynthesis, plant hormone signal transduction, cotyledon vascular tissue pattern formation and spindle organization were found significantly downregulated, while only the cellular response to hypoxia was upregulated in *ddc vs* WT.

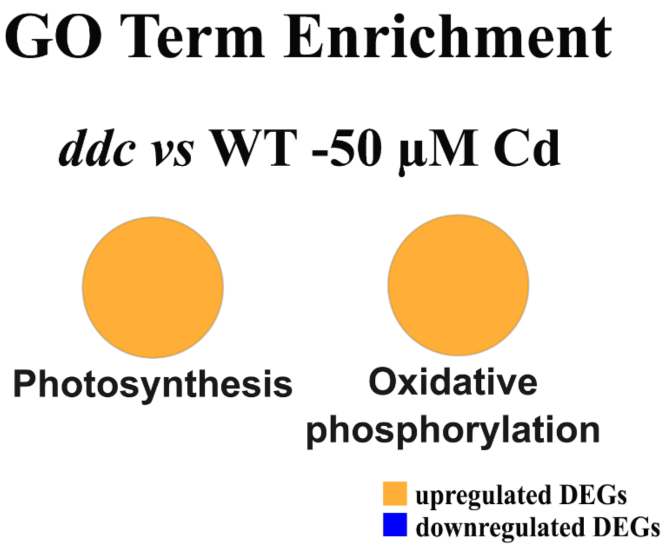

Fig. S5. Gene Enrichment analysis of the DEGs found in the comparison *ddc vs* WT-50 µM Cd. The analysis was performed by using ClueGO plugin of Cytoscape software[12] and the enriched genes were selected on the basis of the calculation of Bonferroni-corrected P-value by using the hypergeometric distribution. The upregulated genes are shown in orange, while the downregulated ones are shown in blue. The size of the nodes reflects the enrichment significance of the terms. Under 50 µM Cd treatment, only a significant upregulation of genetic pathways related to photosynthesis and oxidative phosphorylation pathways was found in *ddc vs* WT.

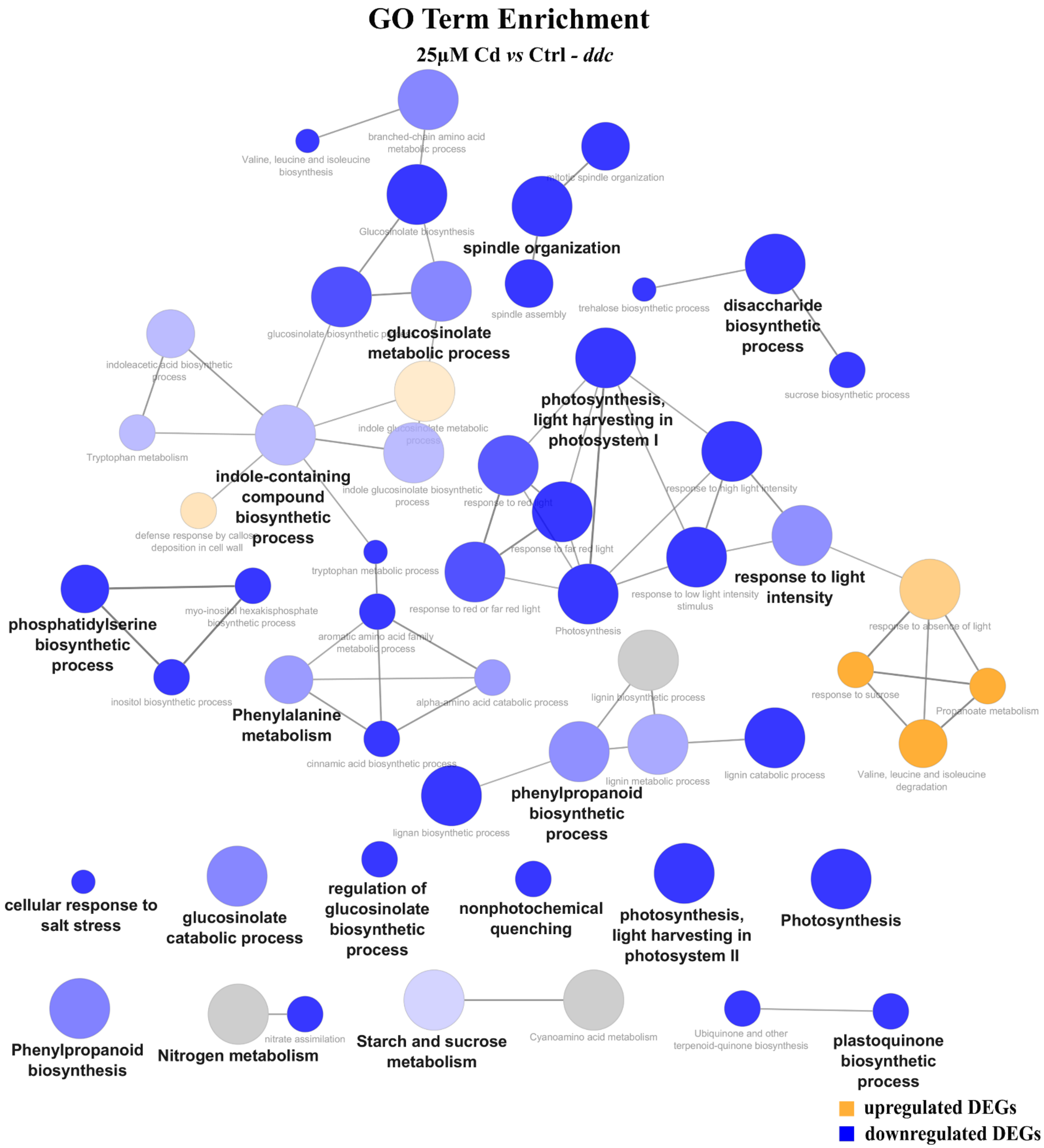

Fig. S6. Gene Enrichment analysis of the DEGs found in the comparison 25 µM Cd *vs* Ctrl-*ddc*. The analysis was performed by using ClueGO plugin of Cytoscape software[12] and the enriched genes were selected on the basis of the calculation of Bonferroni-corrected P-value by using the hypergeometric distribution. The upregulated genes are shown in orange, while the downregulated ones are shown in blue. The size of the nodes reflects the enrichment significance of the terms. 25 µM Cd treatment induced a downregulation of almost all enriched genetic pathways, including photosynthesis, carbohydrate metabolism, glucosinolate metabolism, phenylpropanoid biosynthesis, spindle organization, nonphotochemical quenching, plastoquinone biosynthesis and cellular response to light and salt stress in *ddc* mutant. By contrast, in the same sample was also found a significant upregulation of the genetic pathways related to the response to absence of light and sucrose, the propanoate metabolism and the degradation of valine, leucine and isoleucine.

GO Term Enrichment

50μM Cd *vs* Ctrl - *ddc*

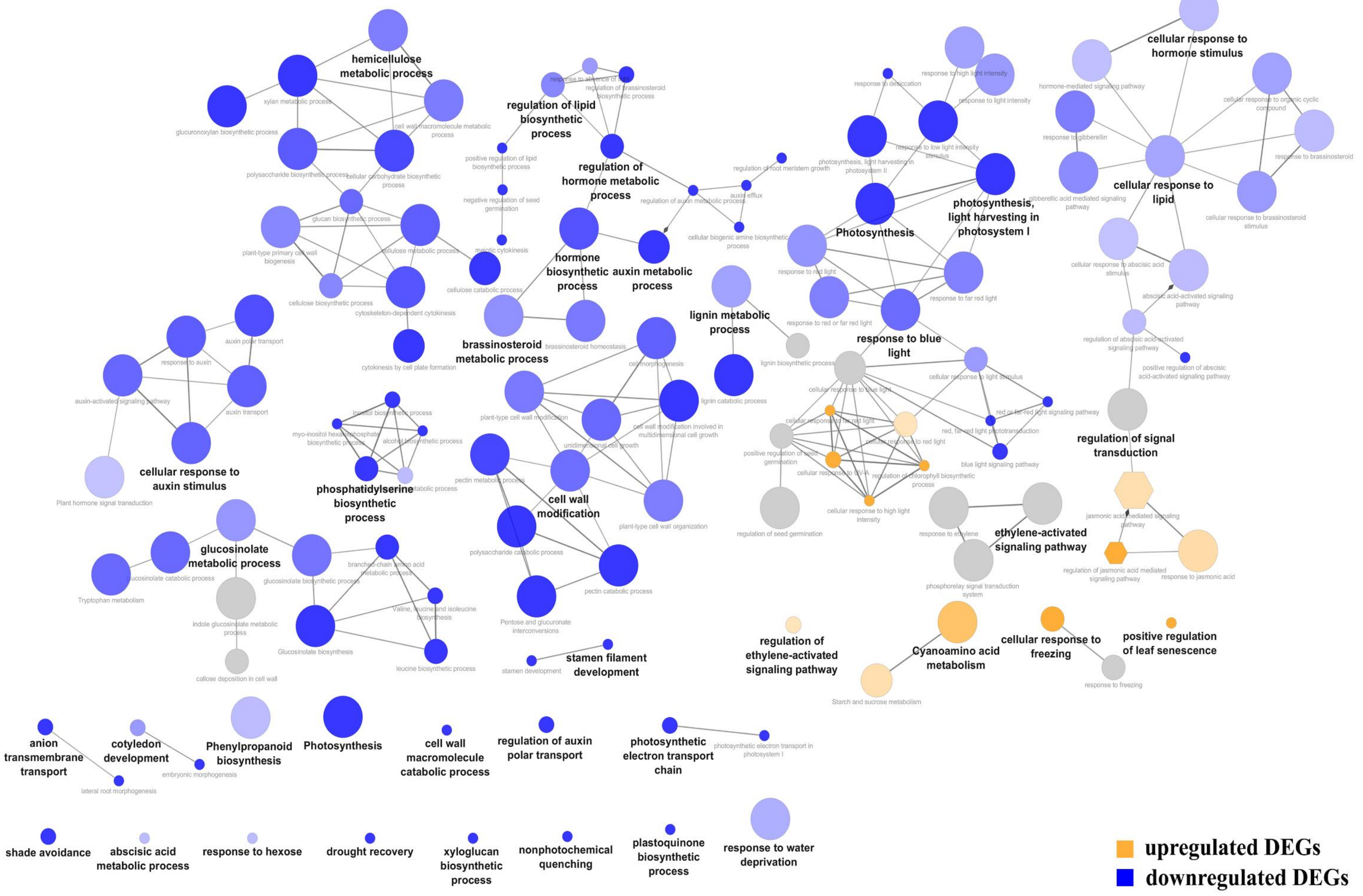

Fig. S7. Gene Enrichment analysis of the DEGs found in the comparison 50 µM Cd *vs* Ctrl-*ddc*. The analysis was performed by using ClueGO plugin of Cytoscape software[12] and the enriched genes were selected on the basis of the calculation of Bonferroni-corrected P-value by using the hypergeometric distribution. The upregulated genes are shown in orange, while the downregulated ones are shown in blue. The size of the nodes reflects the enrichment significance of the terms. 50 µM Cd treatment induced in the *ddc* a significant downregulation of the genetic pathways related to photosynthesis, hemicellulose, lignin and glucosinolate, xiloglucans, phenylpropanoid metabolism, cell wall modifications, brassinosteroids, auxin and abscisic acid metabolism, auxin polar transport, response to blue light, water deprivation and lipids, non-photochemical quenching and cellular response to hormones. Pathways involved in cellular response to freezing, regulation of leaf senescence, response to red light, regulation of chlorophyll biosynthesis, ethylene signalling and its regulation, regulation of signal transduction processes were upregulated.

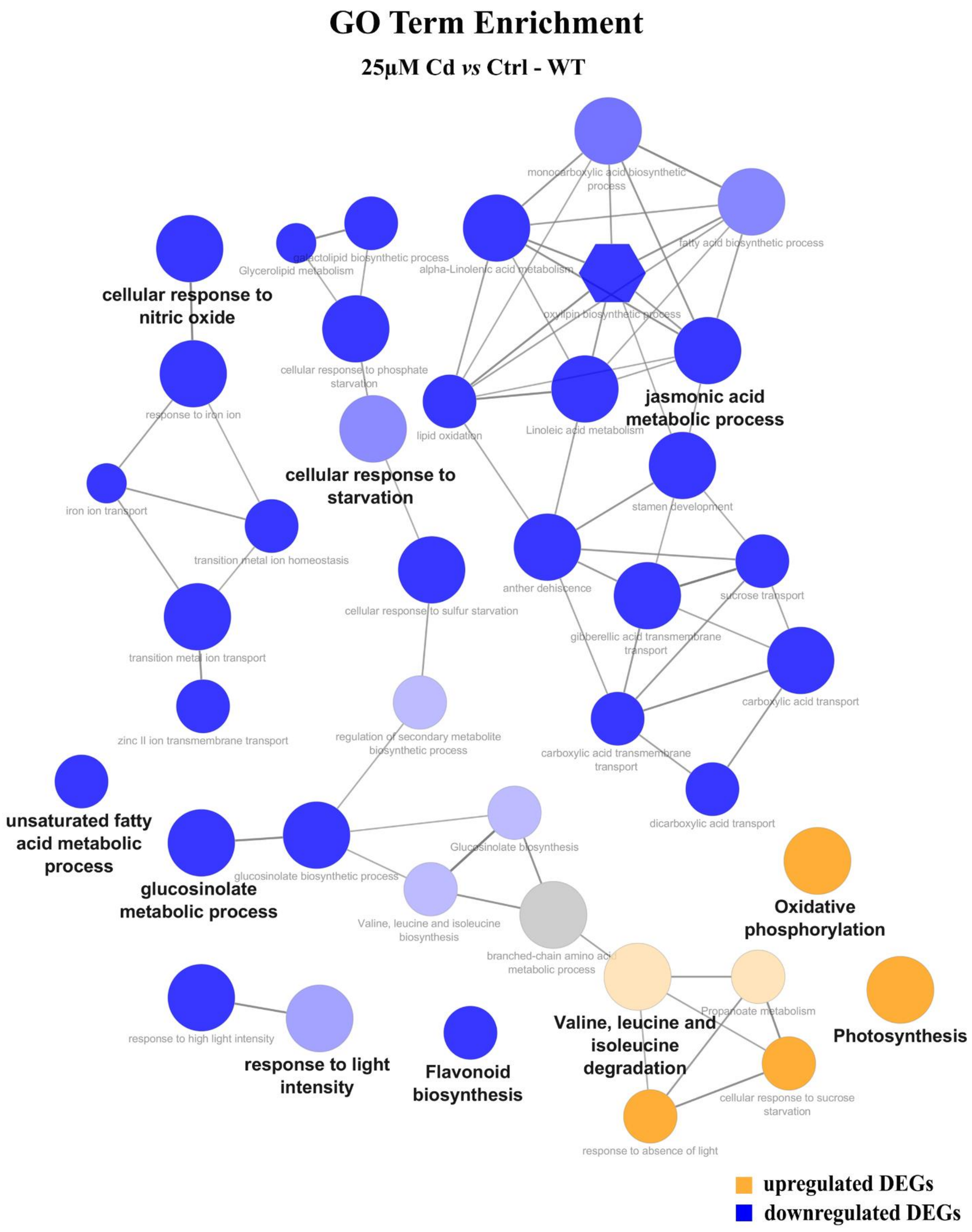

Fig. S8. Gene Enrichment analysis of the DEGs found in the comparison 25 µM Cd *vs* Ctrl-WT. The analysis was performed by using ClueGO plugin of Cytoscape software[12] and the enriched genes were selected on the basis of the calculation of Bonferroni-corrected P-value by using the hypergeometric distribution. The upregulated genes are shown in orange, while the downregulated ones are shown in blue. The size of the nodes reflects the enrichment significance of the terms. 25 µM Cd treatment induced a significant downregulation of genetic pathways related to jasmonic acid, glucosinolate and unsaturated fatty acid metabolism, flavonoid biosynthesis, cellular response to nitric oxide, starvation and light intensity in WT plants while oxidative phosphorylation, photosynthesis and valine, leucine and isoleucine degradation genetic pathways were significantly upregulated.

GO Term Enrichment

50μM Cd *vs* Ctrl - WT

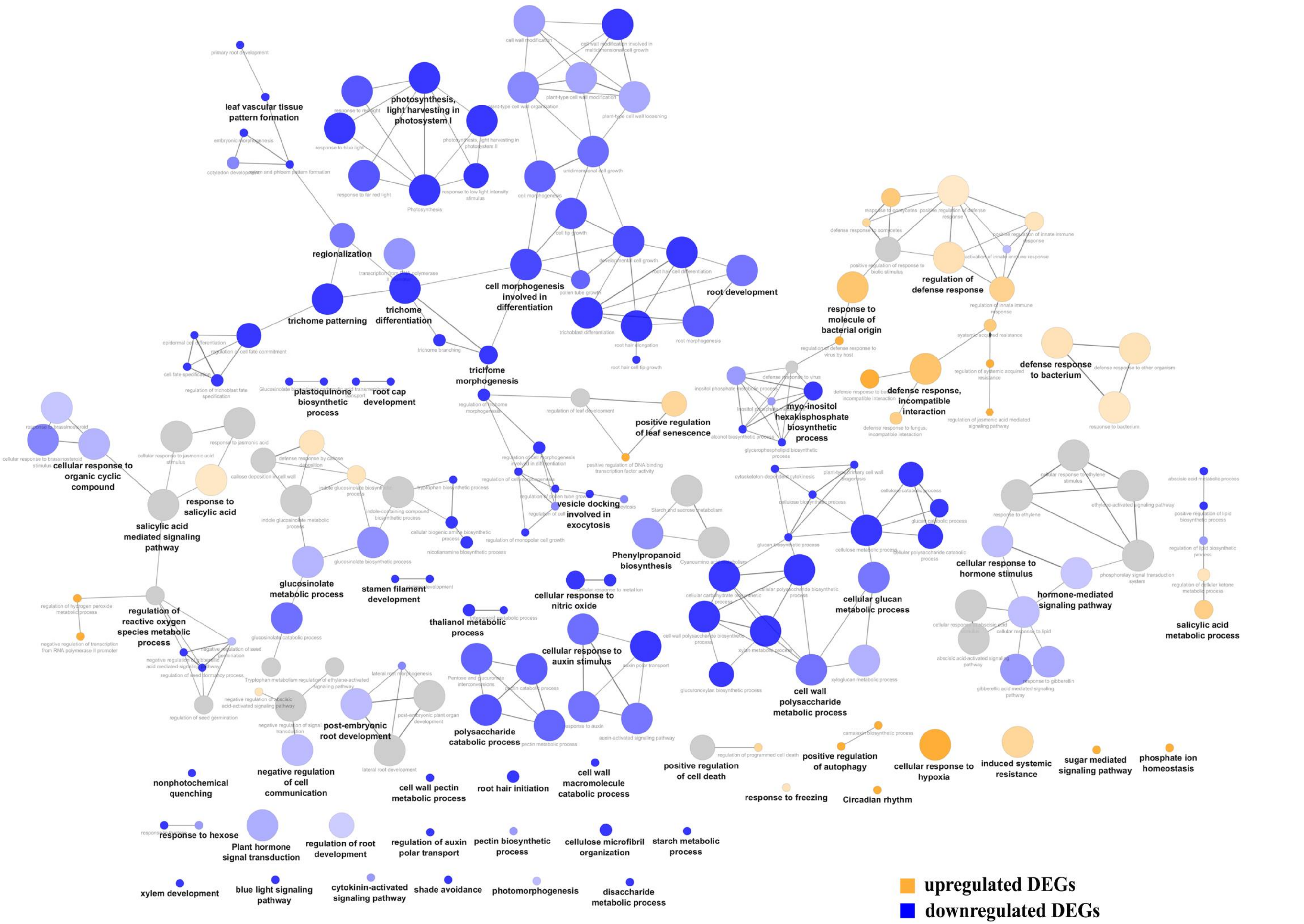

Fig. S9. Gene Enrichment analysis of the DEGs found in the comparison 50 µM Cd *vs* Ctrl-WT. The analysis was performed by using ClueGO plugin of Cytoscape software[12] and the enriched genes were selected on the basis of the calculation of Bonferroni-corrected P-value by using the hypergeometric distribution. The upregulated genes are shown in orange, while the downregulated ones are shown in blue. The size of the nodes reflects the enrichment significance of the terms. Under 50 µM Cd treatment several genetic pathways resulted downregulated in the WT, such as photosynthesis, different carbohydrate metabolism, root development-related processes, exocytosis-related processes, phenylpropanoid biosynthesis and cell walls formation-related processes, glucosinolate metabolism, cellular response to hormone stimulus, plant hormone signal transduction and hormone-mediated signalling pathways, in particular ethylene, abscisic acid, gibberellic acid, auxin and cytokinins-activated signalling. Defense responses were upregulated.

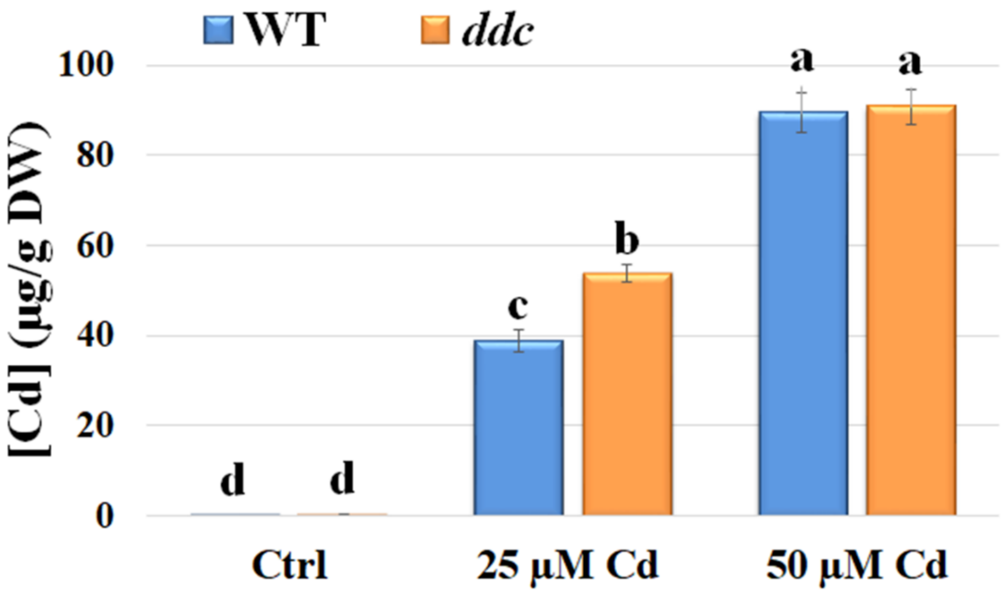

Fig. S10. Cd amount in WT and *ddc* plants of *A. thaliana*, germinated and grown for 21 DAG in long day condition: (i) on growth medium added with 25 or 50 µM Cd; (ii) on growth medium without Cd as control (Ctrl). The results represent the mean value (± SD) of three independent biological replicates (n = 45). Statistical analysis was performed by using two-way ANOVA with Tukey post hoc test ($P \leq 0.05$) after Shapiro–Wilk normality test. Means with the same letter are not significantly different at $P \leq 0.05$.

References

1. Zhang, X. *et al*. Genome-wide High-Resolution Mapping and Functional Analysis of DNA Methylation in Arabidopsis. *Cell* **126**, 1189–1201 (2006).
2. Henderson, I. R. & Jacobsen, S. E. Tandem repeats upstream of the Arabidopsis endogene SDC recruit non-CG DNA methylation and initiate siRNA spreading. Genes Dev. 22, 1597–1606 (2008).
3. Bruno, L. *et al*. In *Arabidopsis thaliana* cadmium impact on the growth of primary root by altering SCR expression and auxin-cytokinin cross-talk. *Front. Plant Sci.* **8**, 1323 (2017).
4. Zhao, Q., Du, Y., Wang, H., Rogers, H. J., Yu, C., Liu, W., Zhao, M., Xie, F. 5-Azacytidine promotes shoot regeneration during Agrobacterium-mediated soybean transformation. *Plant Physiol. Biochem*. **141**, 40-50 (2019).
5. Ye, J. *et al.* Primer-BLAST: a tool to design target-specific primers for polymerase chain reaction. *BMC Bioinformatics* **13**, 134 (2012).
6. Remans, T. *et al.* Normalisation of real-time RT-PCR gene expression measurements in *Arabidopsis thaliana* exposed to increased metal concentrations. *Planta* **227**, 1343–1349 (2008).
7. Rao, X., Lai, D. & Huang, X. A New Method for Quantitative Real-Time Polymerase Chain Reaction Data Analysis. *J. Comput. Biol.* **20**, 703–711 (2013).
8. Fambrini, M., Mariotti, L., Parlanti, S., Salvini, M. & Pugliesi, C. A GRAS-like gene of sunflower (*Helianthus annuus* L.) alters the gibberellin content and axillary meristem outgrowth in transgenic Arabidopsis plants. *Plant Biol.* **17**, 1123–1134 (2015).
9. Scartazza, A., Picciarelli, P., Mariotti, L., Curadi, M., Barsanti, L., Gualtieri, P. The role of *Euglena gracilis* paramylon in modulating xylem hormone levels, photosynthesis and water-use efficiency in *Solanum lycopersicum* L. *Physiol. Plant.* **161**(4), 486-501 (2017).
10. Lulsdorf, M. M. *et al.* Endogenous hormone profiles during early seed development of *C. arietinum and C. anatolicum*. *Plant Growth Regul.* **71**, 191–198 (2013).
11. von Mering, C., Jensen, L.J., Snel, B., Hooper, S.D., Krupp, M., Foglierini, M., Jouffre, N., Huynen, M.A., Bork, P. STRING: known and predicted protein-protein associations, integrated and transferred across organisms. *Nucleic Acids Res.* **33**(Database issue), D433-D437; 10.1093/nar/gki005 (2004).
12. Bindea, G., Mlecnik, B., Hackl, H., Charoentong, P., Tosolini, M., Kirilovsky, A., Fridman, W., Pagès, H. F., Trajanoski, Z., Galon, J. ClueGO: a Cytoscape plug-in to decipher functionally grouped gene ontology and pathway annotation networks. *Bioinformatics* **25**, 1091–1093 (2009).
